# A chromosome-scale genome assembly of *Isatis indigotica*, an important medicinal plant used in traditional Chinese medicine: An *Isatis* genome

**DOI:** 10.1038/s41438-020-0240-5

**Published:** 2020-02-01

**Authors:** Minghui Kang, Haolin Wu, Qiao Yang, Li Huang, Quanjun Hu, Tao Ma, Zaiyun Li, Jianquan Liu

**Affiliations:** 1 Key Laboratory of Bio-Resource and Eco-Environment of Ministry of Education & State Key Laboratory of Hydraulics & Mountain River Engineering, College of Life Sciences, Sichuan University, Chengdu, 610065 China; 2 National Key Laboratory of Crop Genetic Improvement, National Center of Oil Crop Improvement (Wuhan), College of Plant Science and Technology, Huazhong Agricultural University, Wuhan, China; 3 State Key Laboratory of Grassland Agro-Ecosystem, Institute of Innovation Ecology, Lanzhou University, Lanzhou, 730000 China

**Keywords:** Comparative genomics, Medical genomics

## Abstract

*Isatis indigotica* (2*n* = 14) is an important medicinal plant in China. Its dried leaves and roots (called Isatidis Folium and Isatidis Radix, respectively) are broadly used in traditional Chinese medicine for curing diseases caused by bacteria and viruses such as influenza and viral pneumonia. Various classes of compounds isolated from this species have been identified as effective ingredients. Previous studies based on transcriptomes revealed only a few candidate genes for the biosynthesis of these active compounds in this medicinal plant. Here, we report a high-quality chromosome-scale genome assembly of *I*. *indigotica* with a total size of 293.88 Mb and scaffold N50 = 36.16 Mb using single-molecule real-time long reads and high-throughput chromosome conformation capture techniques. We annotated 30,323 high-confidence protein-coding genes. Based on homolog searching and functional annotations, we identified many candidate genes involved in the biosynthesis of main active components such as indoles, terpenoids, and phenylpropanoids. In addition, we found that some key enzyme-coding gene families related to the biosynthesis of these components were expanded due to tandem duplications, which likely drove the production of these major active compounds and explained why *I. indigotica* has excellent antibacterial and antiviral activities. Our results highlighted the importance of genome sequencing in identifying candidate genes for metabolite synthesis in medicinal plants.

## Introduction

The plant family Brassicaceae (Cruciferae) comprises over 330 genera and ~3700 species with a worldwide distribution^1–5^. Numerous crops are derived from this family, including vegetables (*Brassica* and *Raphanu*s), ornamentals (*Matthiola*, *Hesperis*, and *Lobularia*), spices (*Eutrema* and *Armoracia*), and medicines (*Isatis*). Based on sequenced genomes, several model species have been developed for diverse studies, including *Arabidopsis thaliana* for molecular function studies, *Brassica* for polyploidization and whole-genome duplication (WGD) studies, and *Eutrema salsugineum* for abiotic tolerance-related studies. However, genetic biosynthesis of the major active compounds in medicinal plants of this family remains poorly investigated.

*Isatis indigotica* (2*n* = 14) belongs to tribe Isatideae in lineage II of the family^3,6–10^. This species is widely cultivated in China as an important medicinal plant because its dried leaves and roots are used as a traditional Chinese medicine for curing diseases and viruses^11–13^. The major active compounds isolated from this species comprise terpenoids, lignans, and indole alkaloids^14–17^. These compounds were confirmed to have antiviral^18,19^, antibacterial^20^, anti-inflammatory^21,22^, and antileukemia^23,24^ functions. Previous studies based on transcriptomes revealed a few candidate genes involved in the biosynthesis of active compounds in this species^25–28^. However, the limitations of transcriptome quality and integrity hinder the identification of all candidate biosynthesis-related genes.

In the present study, we used single-molecule sequencing combined with high-throughput chromosome conformation capture (Hi-C) technology to assemble the genome and construct the pseudochromosomes of *I. indigotica*. Based on homolog searching and functional annotations, we aimed to identify candidate gene sets involved in the biosynthesis of putative active components. The candidate genes and genomic resources recovered here will be critically important for further experimental verification and artificial syntheses of the active compounds of this medicinal plant in the future.

## Results

### Genome assembly and construction of pseudochromosomes

The genome size, genome repeat size, and heterozygosity rate of *I. indigotica* were estimated using K-mer analysis. The 19-mer frequency of Illumina short reads with the highest peak occurred at a depth of 94. The genome was estimated to be 279.90 Mb in size with 48.99% repeats, and the heterozygosity rate was estimated to be 0.44% (Supplementary Table S3 and Supplementary Fig. S3). In addition, the genome size of *I. indigotica* was estimated to be ~305 Mb based on flow cytometric analyses using *Vigna radiata* as the internal standard (Supplementary Fig. S2).

We sequenced and assembled the genome of *I. indigotica* using single-molecule real-time (SMRT) sequencing technology from Pacific Biosciences (PacBio) and anchored the assembled contigs to seven pseudochromosomes using Hi-C techniques. The final chromosome-scale genome was 293.88 Mb in length with 1199 contigs (contig N50 = 1.18 Mb), a scaffold N50 = 36.17 Mb, and a maximum pseudochromosome length of 38.25 Mb (Table 1, Supplementary Table S4, and Supplementary Fig. S4).

**Table 1:** Statistics for the final genome assembly of *I. indigotica*

	*I. indigotica* genome (PacBio + Hi-C)
Sequencing platform	PacBio Sequel
Assembly size (bp)	293,875,465
GC %	38.18
Number of scaffolds	810
Scaffold N50 size (bp)	36,165,591
Scaffold N90 size (bp)	87,397
Number of contigs	1199
Contig N50 size (bp)	1,176,212
Contig N90 size (bp)	75,736
Gap %	0.01
Longest sequence length (bp)	38,253,781

The completeness of the genome assembly was evaluated using Benchmarking Universal Single-Copy Orthologs (BUSCO)^29^. Of the 1440 plant-specific orthologs, 1416 (98.33%) were identified in the assembly, of which 1400 (97.22%) were considered to be complete (Supplementary Table S5). The assembly base accuracy was also assessed based on Illumina short read mapping. In total, 99.97% of the clean reads were mapped to the genome assembly, and 94.55% of them were properly mapped (Supplementary Table S6). The base error percentage of the genome assembly was estimated to be 0.000081% (Supplementary Table S7). All these evaluations indicate the high completeness, high continuity, and high base accuracy of the present genome assembly.

### Repeat and gene annotations

Repetitive sequences were identified using a combination of ab initio and homology-based approaches. In total, we identified 53.27% of the assembled sequences as repetitive sequences, including 34.67% retrotransposons and 7.37% DNA transposons. Long terminal repeat (LTR) retrotransposons were found to account for 30.09% of the genome (Supplementary Table S8). We annotated protein-coding genes by combining transcriptome-based, homology-based, and ab initio predictions. Finally, we predicted a total of 30,323 genes, of which 5973 had alternatively spliced transcripts. The average transcript length and coding sequence size were 2693 and 1387 bp, respectively, with a mean of 5.50 exons and 1.39 transcripts per gene (Table 2). Overall, 29,522 genes (97.36%) were assigned functions, and 76.16% and 91.69% of these genes had homologies and annotated proteins in the Swiss-Prot and TrEMBL databases. Further functional annotations using InterProScan estimated that 95.86% of the genes contained conserved protein domains, and 87.32% of the genes were classified by Gene Ontology (GO) terms, with 29.41% mapped to known plant biological pathways based on the Kyoto Encyclopedia of Genes and Genomes (KEGG) pathway database (Supplementary Table S9).

**Table 2:** Statistics of predicted protein-coding genes in the *I. indigotica* genome

	*I. indigotica* genome
Number of protein-coding genes	30,323
Number of transcripts	42,061
Average transcript length (bp)	2693.27
Average exon length (bp)	252.24
Average intron length (bp)	215.32
Average number of exons per gene	5.50
Average exon length per gene (bp)	1387.32

### Chromosome structure of *I. indigotica*

**Fig. 1:**
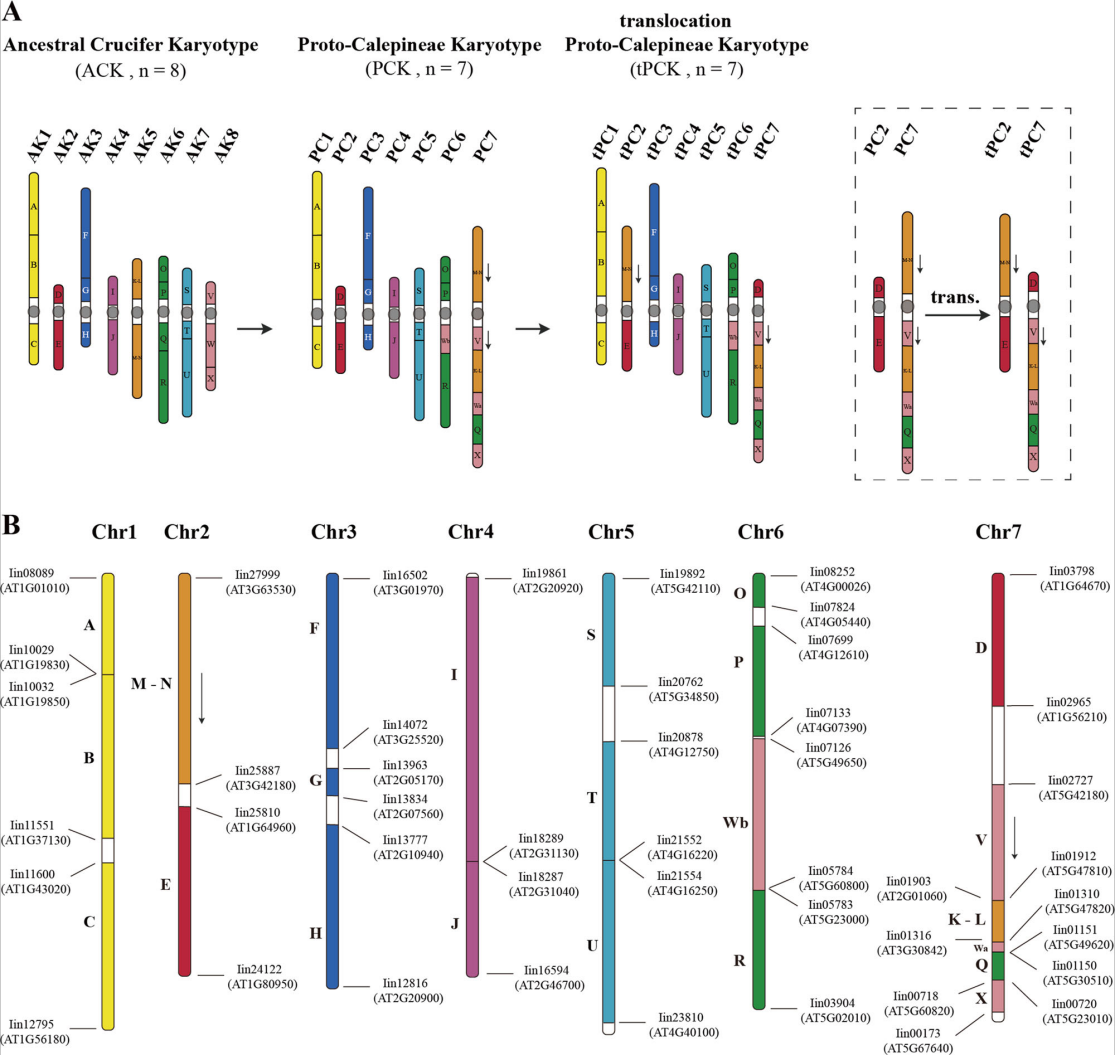
Ancestral Brassicaceae genomes and the distribution of ancestral GBs along the seven pseudochromosomes of *I. indigotica*. **a** The ancestral genomes ACK, PCK, and tPCK, each comprising 22 ancestral GBs. Blocks with opposite orientations relative to that of ACK are represented by downward-pointing arrows. M-N and D GBs are translocated in tPCK chromosomes tPC2 and tPC7 compared with PCK chromosomes PC2 and PC7. The structures were drawn based on previous studies^32–34^. **b** Twenty-two GBs and their positions within the *I. indigotica* genome. Genes of each GB boundary are shown beside the chromosomes with the corresponding *A. thaliana* locus IDs within parentheses based on MCScanX results. The *A. thaliana* GB boundaries were derived from a previous study^34^

Evolution of chromosome structures in Brassicaceae has been traced and established through comparative chromosome painting techniques using BAC probes of the *A. thaliana* genome^4,30^. Using these techniques, Lysak and his colleagues^31^ proposed a model comprising eight chromosomes and 24 genomic blocks (GBs, named from A to X) for comparative genomics and chromosomal analyses based on the Ancestral Crucifer Karyotype (ACK; *n* = 8) concept^31^. This model was further updated to comprise 22 GBs by merging the four GBs to form two new blocks (K-L and M-N), and the GB boundaries defined by *A. thaliana* gene loci were also updated^32^ (Fig. 1a). Six tribes (Calepineae, Coluteocarpeae, Conringieae, Eutremeae, Isatideae, and Sisymbrieae) of expanded lineage II were found to derive from a common ancestor with the Proto-Calepineae Karyotype (PCK; *n* = 7). Among these tribes, three (Eutremeae, Isatideae, and Sisymbrieae) displayed an additional whole-arm translocation in the second and seventh chromosomes (translocation PCK, tPCK; *n* = 7)^32–34^ (Fig. 1a). ACK and PCK shared five similar chromosomes. Thus, they might descend from a common ancestor; alternatively, PCK may have evolved from ACK.

To determine whether the *I. indigotica* genome sequence also supported tPCK structure in Isatideae, we compared the seven pseudochromosomes of *I. indigotica* with the *A. thaliana* genome by LAST and MCScanX. We determined syntenic relationships and constructed the order and orientation of the 22 GBs along the seven pseudochromosomes of the *I. indigotica* genome (Supplementary Figs. S5, S6). Based on the gene intervals of each GB of *A. thaliana*, we determined the corresponding intervals and boundaries of each block in *I. indigotica* and renamed the pseudochromosomes based on Fig. 1a (Fig. 1b and Supplementary Table S10). The *I. indigotica* genome has good collinearity in each GB compared with the *A. thaliana* genome and is consistent with tPCK structure in both order and orientation (Fig. 1 and Supplementary Figs. S5, S6). Furthermore, we carried out sequence alignments between the genomes of *I. indigotica* and the other three species that might also display tPCK structure (*Sisymbrium irio* for Sisymbrieae, *E. salsugineum* for Eutremeae, and *Schrenkiella parvula* for unassigned genera) using LAST. Our analyses suggested that these four species have similar chromosome structures (Supplementary Figs. S7–S9). However, we found obvious inversions in the *S. parvula* genome and low continuity of sequences in the *E. salsugineum* and *S. irio* genomes. These comparisons suggest that the present *I. indigotica* genome was better assembled in terms of both accuracy and continuity than others with tPCK structure.

### Phylogenetic relationships and WGD analyses

**Fig. 2:**
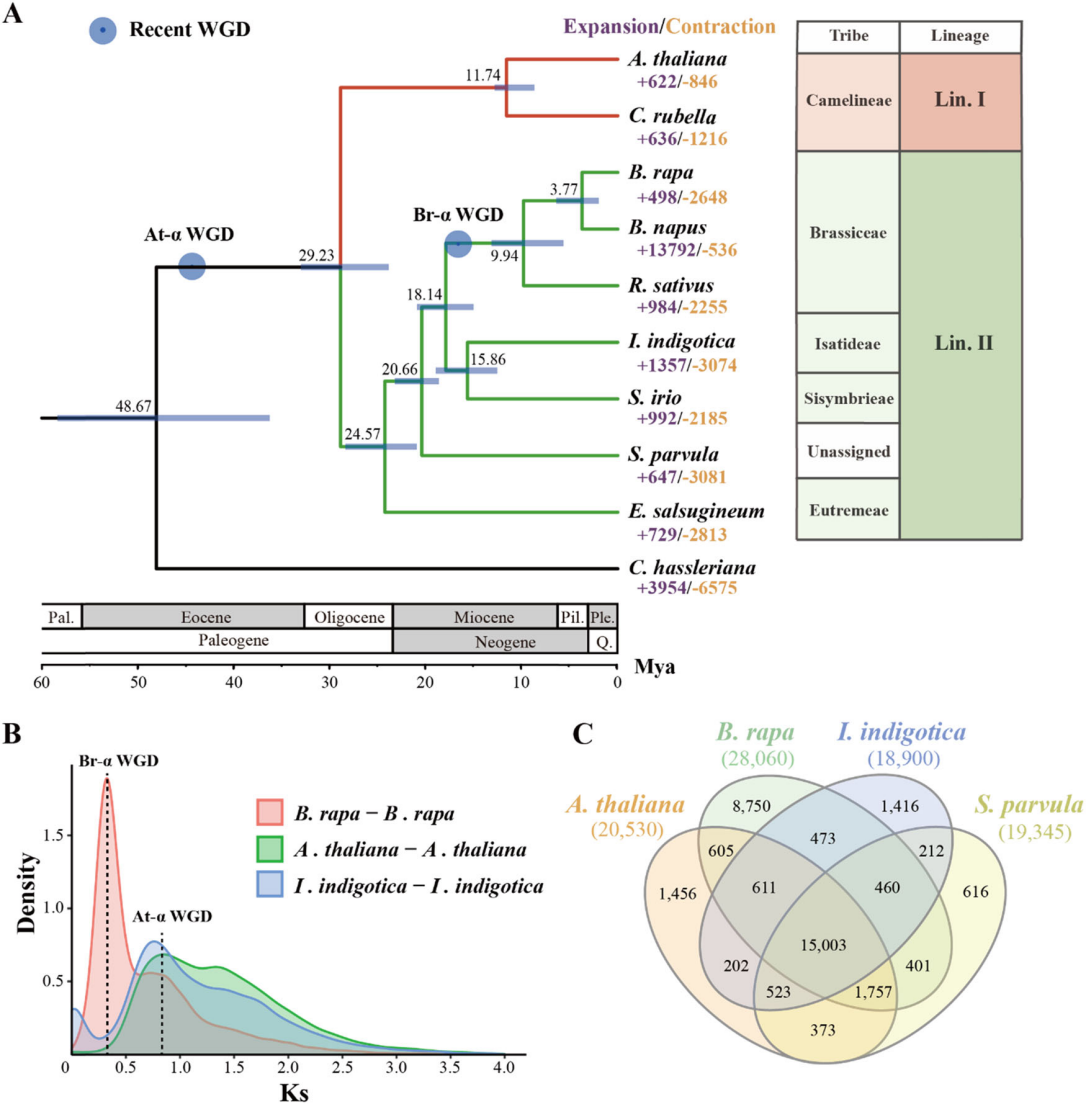
Evolutionary and comparative genomic analyses. **a** The phylogenetic tree of *I. indigotica* and eight other Brassicaceae species with *C. hassleriana* as the outgroup. All branch bootstrap values are 100. Gene family expansions are indicated in purple, while gene family contractions are indicated in light brown. The estimated divergence times (million years ago, Mya) are indicated at each node; bars are the 95% highest probability densities (HPDs). Circles in blue represent recent whole-genome duplication events. **b** Ks value distributions between *B. rapa*, *I. indigotica*, and *A. thaliana*. **c** Orthogroups shared by selected species

We clustered the annotated genes into gene families among *I. indigotica* and eight other Brassicaceae species with *Cleome hassleriana* as the outgroup. A total of 24,382 *I. indigotica* genes (80.41%) clustered into 18,900 gene families, of which 10,826 (57.28%) gene families were shared with nine other species and 896 (4.74%) were *I. indigotica* specific (Fig. 2c and Supplementary Table S11). We selected 822 single-copy gene families among 10 species to construct a phylogenetic tree, which showed that *I. indigotica* was sister to *S. irio*. We further estimated the divergence time between them as 15.86 (12.71–19.20) million years ago (Mya) (Fig. 2a). The relationships of all 10 species are consistent with those from previous phylogenetic analyses^3,6–8,10^.

Then, we used synonymous substitution rates (Ks) between collinear paralogous genes to identify potential WGD events, based on the assumption that the number of silent substitutions per site between two homologous sequences increases in a relatively linear manner with time. A density plot of Ks values for the collinear gene pairs suggested that *I. indigotica* experienced a recent WGD event with a peak value of ~0.76, consistent with At-α-WGD for all Brassicaceae species^8,35,36^. An independent WGD event was identified for *B. rapa* after its divergence from *I. indigotica* at Ks = 0.30–0.34, previously reported as a Brassiceae-specific triplication (Br-α-WGD)^8,37–39^ (Fig. 2b). Whole-genome alignment among the *I. indigotica*, *A. thaliana*, and *B. rapa* genomes carried out by LAST also confirmed the collinear relationship and these WGD events. For each genomic region of *I. indigotica*, we typically found one matching region in *A. thaliana* and three matching regions in *B. rapa*. These comparisons suggest that *I. indigotica* did not experience an independent WGD event after At-α-WGD (Supplementary Figs. S5, S10).

The expansion and contraction of gene families play critical roles in driving phenotypic diversification and enhancing special traits in plants. We discovered 1357 expanded and 3074 contracted gene families in *I. indigotica* relative to *S. irio* (Fig. 2a). Tandem duplication was the main contributor to the gene family expansions. GO enrichment analysis of tandem repeat genes suggested that they were enriched in defense response to virus, indole biosynthetic process, lignin biosynthetic process, flavone synthase activity, and glucosyltransferase activity, some of which might be involved in the biosynthesis of active compounds in *I. indigotica* (Supplementary Table S12). We also performed GO enrichment analysis of the contracted gene families, and the results showed that they were enriched in proton export across plasma membrane, proton-exporting ATPase activity, regulation of stomatal movement, and defense response to other organism (Supplementary Table S13), which are probably related to the environmental adaptation of the species.

### Identification of genes involved in the biosynthetic pathways of active compounds

**Fig. 3:**
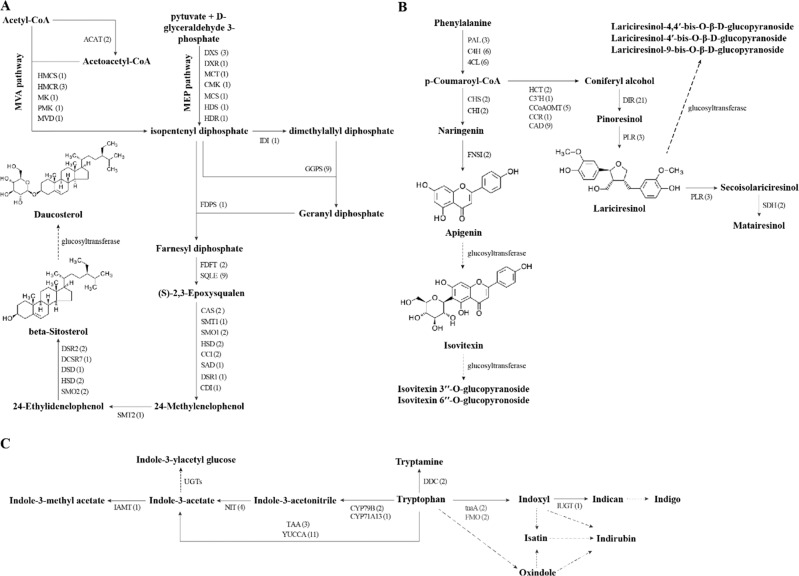
Putative biosynthetic pathways of three main class active compounds in *I. indigotica*. The putative biosynthetic pathways of terpenoids (**a**), phenylpropanoids (**b**), and indole alkaloids (**c**) of active compounds in *I. indigotica*. Values within brackets indicate the numbers of gene copies corresponding to the catalytic genes in the pathways

Based on the KEGG database, GO classification, and the suggested biosynthesis pathways, we used a combined method of homolog searching and functional annotation to identify candidate genes for the biosynthesis of three types of active compounds, namely, terpenoids, phenylpropanoids, and indoles, in *I. indigotica*^14–16,25,40,41^. Sterols are the major terpenoids in *I. indigotica*, mainly comprising β-sitosterol and daucosterol^42^. β-Sitosterol was reported to play a critical role in curing lung inflammation^43^, while daucosterol can inhibit cancer cell proliferation^44^. A total of 59 genes in the present genome, which encoded 31 enzymes, were identified to be involved in terpenoid and sterol biosynthesis (Supplementary Table S14). Based on the functional annotations of these genes, the biosynthesis pathway of β-sitosterol is nearly complete and daucosterol can be further synthesized from β-sitosterol by glucosyltransferases (Fig. 3a). In addition, the intermediate product geranyl diphosphate can be used not only to synthesize sterols but also to produce secologanin for monoterpene indole alkaloids in numerous medicinal plants such as *Catharanthus roseus*^45^. However, we annotated genes only with geraniol 10-hydroxylase activity (GO: 0102811). The lack of other related genes may account for the absence of secologanin and other related monoterpene indole alkaloids in *I. indigotica*.

Phenylpropanoids comprise lignans and flavonoids, with critical roles in anti-inflammatory response, anti-oxidant activity^46^, and attenuation of mammary tumor growth^47^. We annotated 66 genes involved in the biosynthesis of lignans and flavonoids (Supplementary Table S15). The identified putative pathway mainly comprises the biosynthesis of isovitexin and lariciresinol, while their glycosides were further synthesized by glucosyltransferases (Fig. 3b). Indole alkaloids comprise another active component of *I. indigotica*^48–50^ with important anti-influenza, anti-inflammatory, and leukocyte inhibition effects^11,23,51–53^. Based on the KEGG maps and previously suggested pathways^25,54,55^, we identified 32 genes that encoded 11 enzymes involved in the biosynthesis of indole alkaloids (Fig. 3c and Supplementary Table S16). Because of the lack of downstream pathways, other genes for indole alkaloid biosynthesis in *I. indigotica* need further identification. It should be noted that numerous genes involved in the biosynthesis of the three major types of active compounds increased in copy number because of tandem duplication, for example, geranylgeranyl diphosphate synthase, cinnamate 4-hydroxylase, 4-coumarate-CoA ligase, and indole-3-pyruvate monooxygenase (Fig. 3 and Supplementary Tables S14–S16).

## Discussion

Continuity and completeness are important indicators of genome assembly. PacBio-based genome assembly plus error corrections based on Illumina data could greatly improve continuity and completeness^56–59^. Our genome assembly of *I. indigotica* by this strategy showed a highly resolved result with an N50 = 1.22 Mb and longest contig length = 8.99 Mb. In addition, we used Hi-C data to cluster the contigs into seven pseudochromosomes with a final scaffold N50 = 36.17 Mb and longest chromosome length = 38.25 Mb. The completeness and high quality of the present *I. indigotica* genome were further confirmed by BUSCO and comparative chromosome analyses^32^. A total of 97.22% of the genes examined by BUSCO were complete, and the chromosome structure of *I. indigotica* was consistent with the tPCK type.

We constructed the phylogenetic relationships of *I. indigotica* based on genomic data and found that *I. indigotica* of Isatideae is sister to *S. irio* of Sisymbrieae among the sampled species, consistent with the results of previously published phylogenetic analyses^3,6–8,10^. Based on the phylogenetic results, we identified expanded and contracted gene families in *I. indigotica*. The expanded genes in this species were mainly derived from tandem duplications and were obviously enriched in some secondary metabolite pathways. Based on homolog searching and functional annotation in our high-quality genome, we further identified candidate genes for the biosynthesis of three main classes of active compounds in *I. indigotica*: terpenoids, phenylpropanoids, and indole alkaloids. These candidate genes complete or replenish gene sets for biosynthetic pathways of these compounds concentrated in *I. indigotica*^25–28^ (Fig. 3). In addition, we found that in some synthesis steps, the copy number of enzyme-coding genes increased to two or more because of tandem duplications. The increase in copy number may drive the production of major active compounds in *I. indigotica* and account for its excellent antibacterial and antiviral activities because gene expansions are responsible for enhancing a special trait or the origin of a new trait^60–62^.

Overall, in this study, we present a high-quality genome for *I. indigotica*. We further identify or replenish candidate genes for biosynthesis pathways of the active compounds in this medicinal plant. These genes and genomic resources will provide a solid basis for future biosynthesis-related studies.

## Materials and methods

### DNA extraction and genome sequencing

We initially extracted high-quality total DNA from fresh young leaves of a 2-month-old plant artificially cultivated in the greenhouse using the cetyltrimethylammonium bromide method. We used a SMRTbell Template Prep Kit 1.0 (PacBio, Menlo Park, CA, USA) to construct the DNA libraries for PacBio long-read sequencing and sequenced them on a PacBio Sequel system. We obtained a total of four SMRT cells with 39.94 Gb of sequencing data (coverage of 142.71×) from the PacBio Sequel platform and generated a total of 4.30 million subreads with an N50 read length of 14.9 kb (Supplementary Table S1 and Supplementary Fig. S1). We also prepared paired-end Illumina libraries using an Illumina Genomic DNA Sample Preparation Kit and sequenced them on an Illumina HiSeq X Ten system for error correction and K-mer analysis and generated a total of 37.50 Gb of data and 31.79 Gb of clean data (Supplementary Table S1).

### Genome assembly and pseudochromosome construction

We initially estimated the genome size of *I. indigotica* by flow cytometry with *Vigna radiata* as the reference^63^. We then used clean Illumina short reads to calculate K-mers (Illumina DNA short read size of 19 bp) by Jellyfish v.2.2.9^64^ to confirm the genome size. The sequencing depth was estimated by determining the highest peak value of the frequency curve of the K-mer occurrence distribution. We used SMRT Link pipeline v.5.1.0.26412 to process the polymerase reads into subreads with readScore = 0.75 and minSubReadLength = 500 and used Canu v.1.6^65^ to correct errors of the PacBio subreads and assemble the corrected reads into contigs after trimming low-quality bases using WTDBG (https://github.com/ruanjue/wtdbg). We corrected the assembled contigs by using 270 bp PE Illumina data by Pilon v.1.13^66^ and finally obtained a 293.83 Mb contig-scale assembly with a contig N50 of 1.22 Mb. The genome contained 1162 contigs, and the longest contig was 8.99 Mb with a 38.18% GC content. These contigs were further anchored to chromosomes by the Hi-C technique.

We grounded ~3 g of fresh young leaf tissue into powder in liquid nitrogen for Hi-C experiments and constructed a Hi-C library following Louwers et al.^67^ with chromatin extraction and digestion and DNA ligation, purification, and fragmentation. Finally, we obtained a total of 79.43 Gb of clean reads for Hi-C analyses by the Illumina HiSeq X Ten platform. We first carried out a preliminary assembly by splitting contigs into segments of 100 kb on average and mapping the Hi-C data to the contigs using BWA v.0.7.10-r789^68^ in order to correct contig errors. We then used LACHESIS software^69^ with the parameters CLUSTER MIN RE SITES = 22, CLUSTER MAX LINK DENSITY = 2, CLUSTER NONINFORMATIVE RATIO = 2, ORDER MIN N RES IN TRUN = 10, and ORDER MIN N RES IN SHREDS = 10 to cluster and reorder all corrected contigs into pseudochromosomes. We finally adjusted the order and direction of the contigs on the pseudochromosomes by examining their interactions in the Hi-C heatmap. We evaluated the completeness and quality of the final assembled genome through BUSCO v.3.0^29^ tests using gene content from the Embryophyta_odb9 database^29^.

### Repeat annotation

We identified repetitive elements through both RepeatModeler v.1.0.10 and RepeatMasker v.4.0.7^70,71^. RepeatModeler employed RECON and RepeatScout to predict interspersed repeats and then obtained the consensus repeat library. RepeatMasker recovered the repeats in the *I. indigotica* genome through a homology-based repeat search using the ab initio repeat database and Repbase. The overlapping repeats belonging to the same repeat class were combined according to their coordination in the genome. The overlapping repeats belonging to different repeat classes were then split into different types.

### Gene prediction and functional annotation

To improve gene prediction, we further obtained transcriptomes by sequencing high-quality RNA from mixed fresh leaf, flower, and stem tissues and sequenced them by the Illumina HiSeq X Ten platform. We removed adapters and discarded reads with >10% N bases or reads having more than 20% bases of low quality (below 5) using NGS QC Toolkit v.2.3.3^72^ and finally generated 19.87 Gb of clean data. We assembled the de novo and genome-guided transcriptomes with clean reads by Trinity v.2.4.0^73^. We also mapped the RNA-sequencing (RNA-seq) reads to the assembled genome to obtain the mapping rate through HISAT2 v.2.1.0^74^ to evaluate the completeness of the genome.

We run PASA pipeline v.2.1.0^75^ to align the transcripts to the assembled genome to carry out ORF prediction and gene prediction. To train the HMM model for Augustus, we extracted complete, multiexon genes, removed redundant high-identity genes (cut-off all-to-all identity of 70%), and finally generated the best candidate and low-identity gene models for training. We aligned the RNA-seq data to the hard-masked genome assembly by HISAT2^74^ and used bam2hints in Augustus to generate the intron hint file. We used this hint file to carry out ab initio gene prediction by Augustus v.3.2.2^76^. For homologous prediction, the reference protein sequences of *Brassica rapa*, *Brassica napus*, *Raphanus sativus*, *Brassica juncea*, and *Brassica nigra* were downloaded and aligned against the *I. indigotica* genome using TBLASTN v.2.2.31^77^ and searched with an *e* value of 1e^−5^. After filtering low-quality results, gene structure was predicted using GeneWise v.2.4.1^78^. We combined the results from PASA, Augustus and GeneWise to generate the final protein-coding gene set using EVidenceModeler v.1.1.1^75^. To obtain the untranslated regions and alternatively spliced isoforms, we used PASA to update the gff3 file for two rounds and obtain the final gene models.

We annotated the functions of the predicated genes against public databases by NCBI BLAST+ v.2.2.31^77^ with a cut-off *e* value of 1e^−5^ and maximum number of target sequences of 20, including the Swiss-Prot and TrEMBL databases^79^. Best-hit BLAST results were then used to define gene functions. We used InterProScan v.5.25-64.0^80^ to identify motifs and domains by matching against public databases. We identified GO annotations by using Blast2GO v.4.1^81^ according to the blast results and combined them with InterPro GO entries. We mapped the existing GO terms to enzyme codes by Blast2GO and submitted the predicted proteins to the KEGG (Kyoto Encyclopedia of Genes and Genomes) Automatic Annotation Server (KAAS)^82^ to obtain KO numbers for KEGG pathway annotation.

### Gene family and phylogenetic analyses

We used protein sequences of *I. indigotica* and eight other Brassicaceae species (*Arabidopsis thaliana*, *Capsella rubella*, *Brassica rapa*, *Brassica napus*, *Raphanus sativus*, *Schrenkiella parvula*, *Sisymbrium irio*, and *Eutrema salsugineum*) with the outgroup species *Cleome hassleriana* for same-family gene clustering. For genes with alternative splicing variants, the longest transcript was selected to represent the gene. Similarities between sequence pairs were calculated using BLASTP v.2.2.31^77^ with a cut-off *e* value of 1e^−5^. Additionally, OrthoMCL v.2.0.9 was used with default parameters to assess gene family membership based on overall gene similarity combined with Markov Chain Clustering (MCL) v.14-137^83^.

We extracted single-copy orthologous genes from the ten species by OrthoMCL and aligned the resulting protein sequences by MAFFT v.7.313^84^. Then, we used Gblocks v.0.91b^85^ to extract the conserved sites of multiple sequence alignments and constructed a phylogenetic tree by RAxML v.8.2.11^86^. We used *C. hassleriana* as an outgroup and performed 1000 bootstrap analyses to test the robustness of each branch. We used the Bayesian relaxed molecular clock approach in MCMCTREE of PAML v.4.9e^87^ to estimate divergence time. We calibrated this tree based on the estimated divergence times in the TimeTree database^88^ for *C. hassleriana*–*A. thaliana* (35–59 Mya), *A. thaliana*–*C. rubella* (7.4–12.8 Mya), *B. rapa–S. parvula* (19.3–28.6 Mya), and *B. rapa–A. thaliana* (23.4–33.5 Mya).

Gene families that had undergone expansion or contraction were identified in the eight sequenced species using CAFE^89^. The CAFE parameters included a *p* value threshold = 0.05 and automatic searching for the *λ* value. The algorithm in CAFE takes a matrix of gene family sizes in extant species as input and uses a probabilistic graphical model to ascertain the rate and direction of changes in gene family size across a given phylogenetic tree.

### WGD analysis and identification of tandemly repeated genes

To examine WGD in *I. indigotica* and *B. rapa*, we extracted all homologous proteins between these two species and *A. thaliana* using an all-to-all search in BLASTP v.2.2.31^77^ with an *e* value cut-off of 1e^−9^. We used MCScanX^90^ with default parameters to identify collinear blocks, each containing at least five collinear gene pairs. To infer WGD events, we used the downstream MCScanX script add_ka_and_ks_to_collinearity.pl to calculate the Ks values between collinear genes among these three genomes. We further performed whole-genome alignment of the three species by LAST v.946^91^ and constructed a dot plot by the downstream program last-dotplot.

Identification of tandem repeat genes in the *I. indigotica* genome was based on three criteria: (1) two or more genes had more than 70% identity and 70% coverage according to BLASTP; (2) the pairwise gene distance was <100 kb; and (3) there were no more than 10 genes lying between the repeat genes on a single scaffold^92^. The genes identified in this way were subjected to functional analysis using GO enrichment.

## Supplementary information

Supplementary Information of A chromosome-scale genome assembly of Isatis indigotica, one important medicinal plant used in traditional Chinese medicine

## Data Availability

The PacBio long reads and Illumina short reads were uploaded to the NCBI SRA database under BioProject PRJNA549758. The final chromosome-scale genome assembly was submitted to the NCBI with accession number VHIU00000000. The genome fasta and gff3 files were uploaded to Figshare.

## References

[1] Rollins, R. C. *The Cruciferae of Continental North America: Systematics of the Mustard Family from the Arctic to Panama* (Stanford University Press, 1993).

[2] Al-Shehbaz, I. A. & Mummenhoff, K. Transfer of the South African genera Brachycarpaea, Cycloptychis, Schlechteria, Silicularia, and Thlaspeocarpa to Heliophila (Brassicaceae). *Novon* **15**, 385–389 (2005).

[3] Al-Shehbaz I, Beilstein MA, Kellogg E (2006). Systematics and phylogeny of the Brassicaceae (Cruciferae): an overview. Plant Syst. Evol..

[4] Lysak, M. A. & Koch, M. A. Phylogeny, genome, and karyotype evolution of crucifers (Brassicaceae). In Schmidt, R. & Bancroft, I. (eds) *Genetics and Genomics of the Brassicaceae* 1–31 (Springer, New York, New York, USA, 2011).

[5] Warwick SI, Francis A, Al-Shehbaz IA (2006). Brassicaceae: species checklist and database on CD-Rom. Plant Syst. Evol..

[6] Beilstein M, Al-Shehbaz I, Kellogg E (2006). Brassicaceae phylogeny and trichome evolution. Am. J. Bot..

[7] Beilstein MA, Al-Shehbaz IA, Sarah M, Kellogg EA (2008). Brassicaceae phylogeny inferred from phytochrome A and ndhF sequence data: tribes and trichomes revisited. Am. J. Bot..

[8] Franzke A, Lysak MA, Al-Shehbaz IA, Koch MA, Mummenhoff K (2011). Cabbage family affairs: the evolutionary history of Brassicaceae. Trends Plant Sci..

[9] Guo X (2017). Plastome phylogeny and early diversification of Brassicaceae. BMC Genomics.

[10] Nikolov LA (2019). Resolving the backbone of the Brassicaceae phylogeny for investigating trait diversity. N. Phytol..

[11] Chang, S. J., Chang, Y. C., Lu, K. Z., Tsou, Y. Y. & Lin, C. W. Antiviral activity of *Isatis indigotica* extract and its derived indirubin against Japanese encephalitis virus. *Evid. Based Complement Alternat. Med.* **2012**, 925830 (2012). 10.1155/2012/925830 PMC3405817 22911608

[12] Liu S (2000). Antiviral action of Radix Isatidis and Folium Isatidis from different germplasm against influenza A virus. Acad. J. Second Mil. Med. Univ..

[13] Du Z, Liu H, Zhang Z, Li P (2013). Antioxidant and anti-inflammatory activities of Radix Isatidis polysaccharide in murine alveolar macrophages. Int. J. Biol. Macromol..

[14] Chen M (2012). Alkaloids from the root of *Isatis indigotica*. J. Nat. Prod..

[15] Deng X, Gao G, Zheng S, Li F (2008). Qualitative and quantitative analysis of flavonoids in the leaves of *Isatis indigatica* Fort. by ultra-performance liquid chromatography with PDA and electrospray ionization tandem mass spectrometry detection. J. Pharm. Biomed..

[16] Li B (2005). Phenylpropanoids isolated from tetraploid roots of *Isatis indigotica*. Chin. Tradit. Herbal Drugs.

[17] Zhou W, Zhang XY (2013). Research progress of Chinese herbal medicine *Radix isatidis* (banlangen). Am. J. Chin. Med..

[18] Lin CW (2005). Anti-SARS coronavirus 3C-like protease effects of *Isatis indigotica* root and plant-derived phenolic compounds. Antivir. Res..

[19] Sun DD, Dong WW, Li X, Zhang HQ (2010). Indole alkaloids from the roots of *Isatis ingigotica* and their antiherpes simplex virus type 2 (HSV-2) activity in vitro. Chem. Nat. Compd..

[20] Xia X, Xiao J, Shi G, W. D (2007). Function research of resistance to Salmonella Typhimurium infection using Banlangen polysaccharide. Med. J. Wuhan Univ..

[21] Ho YL, Chang YS (2002). Studies on the antinociceptive, anti-inflammatory and antipyretic effects of *Isatis indigotica* root. Phytomedicine.

[22] During A, Debouche C, Raas T, Larondelle Y (2012). Among plant lignans, pinoresinol has the strongest antiinflammatory properties in human intestinal Caco-2 cells. J. Nutr..

[23] Hoessel R (1999). Indirubin, the active constituent of a Chinese antileukaemia medicine, inhibits cyclin-dependent kinases. Nat. Cell Biol..

[24] Molina P (2001). Inhibition of leukocyte functions by the alkaloid isaindigotone from *Isatis indigotica* and some new synthetic derivatives. J. Nat. Prod..

[25] Chen J (2013). Biosynthesis of the active compounds of *Isatis indigotica* based on transcriptome sequencing and metabolites profiling. BMC Genomics.

[26] Gai QY (2019). Elicitation of *Isatis tinctoria* L. hairy root cultures by salicylic acid and methyl jasmonate for the enhanced production of pharmacologically active alkaloids and flavonoids. Plant Cell.

[27] Lu BB (2006). Cloning and characterization of a differentially expressed phenylalanine ammonialyase gene (IiPAL) after genome duplication from tetraploid *Isatis indigotica* Fort. J. Integr. Plant Biol..

[28] Hu Y (2011). Isolation and characterization of a gene encoding cinnamoyl-CoA reductase from *Isatis indigotica* Fort. Mol. Biol. Rep..

[29] SimãO FA, Waterhouse RM, Panagiotis I, Kriventseva EV, Zdobnov EM (2015). BUSCO: assessing genome assembly and annotation completeness with single-copy orthologs. Bioinformatics.

[30] Mandáková, T. & Lysak, M. A. Painting of *Arabidopsis* chromosomes with chromosome-specific BAC clones. *Curr. Protocols Plant Biol.* **1**, 359–371 (2016). 10.1002/cppb.20022 30775864

[31] Schranz ME, Lysak MA, Mitchell-Olds T (2006). The ABC’s of comparative genomics in the Brassicaceae: building blocks of crucifer genomes. Trends Plant Sci..

[32] Lysak MA, Mandáková T, Schranz ME (2016). Comparative paleogenomics of crucifers: ancestral genomic blocks revisited. Curr. Opin. Plant Biol..

[33] Terezie M, Lysak MA (2008). Chromosomal phylogeny and karyotype evolution in *x* = 7 crucifer species (Brassicaceae). Plant Cell.

[34] Cheng F (2013). Deciphering the diploid ancestral genome of the mesohexaploid *Brassica rapa*. Plant Cell.

[35] Bowers JE, Chapman BA, Rong J, Paterson AH (2003). Unravelling angiosperm genome evolution by phylogenetic analysis of chromosomal duplication events. Nature.

[36] Barker MS, Heiko V, Eric SM (2009). Paleopolyploidy in the Brassicales: analyses of the cleome transcriptome elucidate the history of genome duplications in *Arabidopsis* and other Brassicales. Genome Biol. Evol..

[37] Lagercrantz U, Lydiate DJ (1996). Comparative genome mapping in Brassica. Genetics.

[38] Schranz ME, Mohammadin S, Edger PP (2012). Ancient whole genome duplications, novelty and diversification: the WGD Radiation Lag-Time Model. Curr. Opin. Plant Biol..

[39] Tank DC (2015). Nested radiations and the pulse of angiosperm diversification: increased diversification rates often follow whole genome duplications. N. Phytol..

[40] Mohn T, Plitzko I, Hamburger M (2009). A comprehensive metabolite profiling of *Isatis tinctoria* leaf extracts. Phytochemistry.

[41] Yang L (2014). Indole alkaloids from the roots of *Isatis indigotica* and their inhibitory effects on nitric oxide production. Fitoterapia.

[42] He LW, Li X, Chen JW (2005). Research progress of antiviral active components of *Radix Isatidis*. Inform. Trad. Chin. Med..

[43] Yuk JE (2007). Effects of lactose-β-sitosterol and β-sitosterol on ovalbumin-induced lung inflammation in actively sensitized mice. Int. Immunopharmacol..

[44] Zhao C (2015). Daucosterol inhibits cancer cell proliferation by inducing autophagy through reactive oxygen species-dependent manner. Life Sci..

[45] Kellner F (2015). Genome‐guided investigation of plant natural product biosynthesis. Plant J..

[46] Lv H (2016). Isovitexin exerts anti-inflammatory and anti-oxidant activities on lipopolysaccharide-induced acute lung injury by inhibiting MAPK and NF-κB and activating HO-1/Nrf2 pathways. Int. J. Biol. Sci..

[47] Saarinen NM (2010). Dietary lariciresinol attenuates mammary tumor growth and reduces blood vessel density in human MCF-7 breast cancer xenografts and carcinogen-induced mammary tumors in rats. Int. J. Cancer.

[48] Meng LJ (2017). Diglycosidic indole alkaloid derivatives from an aqueous extract of *Isatis indigotica* roots. J. Asian Nat. Prod. Res..

[49] Wu Y (2011). Novel indole C-glycosides from *Isatis indigotica* and their potential cytotoxic activity. Fitoterapia.

[50] Liu YF (2015). Antiviral glycosidic bisindole alkaloids from the roots of *Isatis indigotica*. J. Asian Nat. Prod. Res..

[51] Mak NK (2004). Inhibition of RANTES expression by indirubin in influenza virus-infected human bronchial epithelial cells. Biochem. Pharmacol..

[52] Kunikata T (2000). Indirubin inhibits inflammatory reactions in delayed-type hypersensitivity. Eur. J. Pharmacol..

[53] Salvini M (2008). Alpha-tryptophan synthase of *Isatis tinctoria*: gene cloning and expression. Plant Physiol. Biochem..

[54] Hsu Tammy M, Welner Ditte H, Russ Zachary N, Cervantes Bernardo, Prathuri Ramya L, Adams Paul D, Dueber John E (2018). Employing a biochemical protecting group for a sustainable indigo dyeing strategy. Nature Chemical Biology.

[55] Sheng H (2010). Altering regioselectivity cytochrome P450 BM-3 saturation mutagenes is for the biosynthesis of indirubin. J. Mol. Catal. B.

[56] Korlach J (2017). De novo PacBio long-read and phased avian genome assemblies correct and add to reference genes generated with intermediate and short reads. GigaScience.

[57] Zhang L (2018). Improved *Brassica rapa* reference genome by single-molecule sequencing and chromosome conformation capture technologies. Horticult. Res..

[58] Chen J (2019). Liriodendron genome sheds light on angiosperm phylogeny and species–pair differentiation. Nat. Plants.

[59] Hu, Y. et al. *Gossypium barbadense* and *Gossypium hirsutum* genomes provide insights into the origin and evolution of allotetraploid cotton. *Nat. Genet*. **51**, 739–748 (2019). 10.1038/s41588-019-0371-5 30886425

[60] Chae L, Kim T, Nilo-Poyanco R, Rhee SY (2014). Genomic signatures of specialized metabolism in plants. Science.

[61] Kliebenstein DJ (2008). A role for gene duplication and natural variation of gene expression in the evolution of metabolism. PLoS ONE.

[62] Kliebenstein DJ, Lambrix VM, Reichelt M, Gershenzon J, Mitchell-Olds T (2001). Gene duplication in the diversification of secondary metabolism: tandem 2-oxoglutarate-dependent dioxygenases control glucosinolate biosynthesis in *Arabidopsis*. Plant Cell.

[63] Kang YJ (2014). Genome sequence of mungbean and insights into evolution within *Vigna* species. Nat. Commun..

[64] Marçais G, Kingsford C (2011). A fast, lock-free approach for efficient parallel counting of occurrences of k-mers. Bioinformatics.

[65] Koren S (2017). Canu: scalable and accurate long-read assembly via adaptive k-mer weighting and repeat separation. Genome Res..

[66] Walker BJ (2014). Pilon: an integrated tool for comprehensive microbial variant detection and genome assembly improvement. PLoS ONE.

[67] Louwers M, Splinter E, Driel RV, Laat WD, Stam M (2009). Studying physical chromatin interactions in plants using Chromosome Conformation Capture (3C). Nat. Protoc..

[68] Li H, Durbin R (2009). Fast and accurate short read alignment with Burrows–Wheeler transform. Bioinformatics.

[69] Burton JN (2013). Chromosome-scale scaffolding of de novo genome assemblies based on chromatin interactions. Nat. Biotechnol..

[70] Chen N (2004). Using RepeatMasker to identify repetitive elements in genomic sequences. Curr. Protoc. Bioinformatics.

[71] Price AL, Jones NC, Pevzner PA (2005). De novo identification of repeat families in large genomes. Bioinformatics.

[72] Patel RK, Mukesh J (2012). NGS QC Toolkit: a toolkit for quality control of next generation sequencing data. PLos ONE.

[73] Grabherr, M. G. et al. Trinity: reconstructing a full-length transcriptome without a genome from RNA-seq data. *Nat. Biotechnol.* **29**, 644–652 (2011). 10.1038/nbt.1883 PMC3571712 21572440

[74] Daehwan K, Ben L, Salzberg SL (2015). HISAT: a fast spliced aligner with low memory requirements. Nat. Methods.

[75] Haas BJ (2008). Automated eukaryotic gene structure annotation using EVidenceModeler and the program to assemble spliced alignments. Genome Biol..

[76] Mario S, Rasmus S, Stephan W, Burkhard M (2004). AUGUSTUS: a web server for gene finding in eukaryotes. Nucleic Acids Res..

[77] Camacho C (2009). BLAST+: architecture and applications. BMC Bioinformatics.

[78] Birney E, Clamp M, Durbin R (2004). GeneWise and genomewise. Genome Res..

[79] Bairoch A, Apweiler R (2000). The SWISS-PROT protein sequence database and its supplement TrEMBL in 2000. Nucleic Acids Res..

[80] Zdobnov EM, Apweiler R (2001). InterProScan—an integration platform for the signature-recognition methods in InterPro. Bioinformatics.

[81] Conesa, A. & Götz, S. Blast2GO: a comprehensive suite for functional analysis in plant genomics. *Int. J. Plant Genomics* **2008**, 619832 (2008). 10.1155/2008/619832 PMC2375974 18483572

[82] Moriya Y, Itoh M, Okuda S, Yoshizawa AC, Kanehisa M (2007). KAAS: an automatic genome annotation and pathway reconstruction server. Nucleic Acids Res..

[83] Li L, Stoeckert CJ, Roos DS (2003). OrthoMCL: identification of ortholog groups for eukaryotic genomes. Genome Res..

[84] Katoh K, Standley DM (2013). MAFFT multiple sequence alignment software version 7: improvements in performance and usability. Mol. Biol. Evol..

[85] Castresana J (2000). Selection of conserved blocks from multiple alignments for their use in phylogenetic analysis. Mol. Biol. Evol..

[86] Stamatakis A (2014). RAxML version 8: a tool for phylogenetic analysis and post-analysis of large phylogenies. Bioinformatics.

[87] Yang Z (2007). PAML 4: phylogenetic analysis by maximum likelihood. Mol. Biol. Evol..

[88] Kumar S, Stecher G, Suleski M, Hedges SB (2017). TimeTree: a resource for timelines, timetrees, and divergence times. Mol. Biol. Evol..

[89] Han MV, Thomas GWC, Jose LM, Hahn MW (2013). Estimating gene gain and loss rates in the presence of error in genome assembly and annotation using CAFE 3. Mol. Biol. Evol..

[90] Wang Y (2012). MCScanX: a toolkit for detection and evolutionary analysis of gene synteny and collinearity. Nucleic Acids Res..

[91] Kiełbasa SM, Wan R, Sato K, Horton P, Frith MC (2011). Adaptive seeds tame genomic sequence comparison. Genome Res..

[92] Hanada K, Zou C, Lehti-Shiu MD, Shinozaki K, Shiu SH (2008). Importance of lineage-specific expansion of plant tandem duplicates in the adaptive response to environmental stimuli. Plant Physiol..

