# Supplementary material for: A chromosome-scale genome assembly of Isatis indigotica, an important medicinal plant used in traditional Chinese medicine: An Isatis genome
Source: Hortic Res. 2020 Feb 1;7:18. doi: 10.1038/s41438-020-0240-5 (PMC6994597; doi:10.1038/s41438-020-0240-5)
Supplement: Supplementary file 1 — Supplementary Information of A chromosome-scale genome assembly of Isatis indigotica, one important medicinal plant used in traditional Chinese medicine [file 41438_2020_240_MOESM1_ESM.docx]

**Tables**

**Supplementary Table S1. Summary of DNA sequencing data.**

| **Read type** | **Number of clean reads / subreads** | **Insert size (bp)** | **Total data (G**b) | **Read length / Mean subread length (N50) (bp)** | **Sequence coverage**^1^ (X) |
| --- | --- | --- | --- | --- | --- |
| **Illumina pair-end reads** | 106,160,329 | 270 | 31.79 | 2 x 150 | 113.60 |
| **Hi-C pair-end reads** | 132,725,606 | 300 | 79.43 | 2 x 150 | 283.79 |
| **Pacbio subreads** | 4,299,248 | - | 39.94 | 9,291 (14,892) | 142.71 |

^1^Depth was calculated under the estimate of a genome size of 279.90Mb.

**Supplementary Table S2. Summary of RNA sequencing data.**

| **Tissue** | **Read type** | **Number of reads** | **Total data (G**b) | **Read length (bp)** | **Mapping rate (%)** |
| --- | --- | --- | --- | --- | --- |
| Leaf+Stem+Flower | Illumina paired-end | 66,416,973 | 19.87 | 2 x 150 | 93.31 |

**Supplementary Table S3. K-mer analysis of the *I. indigotica* genome by using K-mer=19.**

| **K-mer** | **K-mer number** | **K-mer Depth (X)** | **Genome Size（Mb）** | **Heterozygous Ratio(%)** | **Repeat(%)** |
| --- | --- | --- | --- | --- | --- |
| 19 | 26,559,599,210 | 94 | 279.90 | 0.44 | 48.99 |

**Supplementary Table S4. Statistics of *I. indigotica* genome after assembly by Hi-C data.**

| **Group** | **Sequence Number**^†^ | **Sequence Length (bp)**^1^ |
| --- | --- | --- |
| Lachesis Group 0 | 176 | 45,173,941 |
| Lachesis Group 1 | 131 | 41,959,486 |
| Lachesis Group 2 | 122 | 41,928,255 |
| Lachesis Group 3 | 167 | 40,875,629 |
| Lachesis Group 4 | 164 | 38,824,557 |
| Lachesis Group 5 | 163 | 43,738,998 |
| Lachesis Group 6 | 101 | 37,126,907 |
| Total Sequences Clustered (%) | 1,024 (85.4) | 289,627,773(98.57) |
| Total Sequences Ordered and Oriented (%) | 396 (38.67) | 250,784,150(86.59) |

^1^Sequence number and length was calculated with all clustered sequences > 1 Kb.

**Supplementary Table S5. Summary of BUSCO analysis about** ***I. indigotica*** **genome assembly.**

|  | **Total BUSCO groups searched** | **Complete single-copy BUSCOs** | **Complete duplicated BUSCOs** | **Fragmented BUSCOs** | **Missing BUSCOs** | **Complete BUSCOs Percentage (%)** |
| --- | --- | --- | --- | --- | --- | --- |
| 1. ***indigotica* genome** | 1,440 | 1,300 | 100 | 16 | 24 | 97.2 |

**Supplemental Table S6.** **Statistics of reads mapping rate of *I. indigotica* genome assembly.**

| **Library** | **Total reads** | **Mapped reads** | **Mapped(%)** | **Properly mapped reads** | **Properly_mapped(%)** |
| --- | --- | --- | --- | --- | --- |
| 270bp | 207,077,856 | 207,023,685 | 99.97 | 195,792,596 | 94.55 |

**Supplemental Table S7.** **Statistics of error base percentage in *I. indigotica* genome assembly.**

| **Contig**  **length (bp)** | **Correct base**  **number (bp)** | **Error base**  **number (bp)** | **Error base**  **percentage (%)** |
| --- | --- | --- | --- |
| 293,836,565 | 293,834,196 | 2,369 | 0.000081 |

**Supplementary Table S8. Classification of repetitive elements in the *I. indigotica* genome.**

| **Type** | **Repeat size (bp)** | **number of elements** | **% of genome** |
| --- | --- | --- | --- |
| **DNA** | 21,656,011 | 79,285 | 7.37 |
| **LINE** | 12,251,623 | 26,285 | 4.17 |
| **SINE** | 1,199,307 | 4,107 | 0.41 |
| **LTR** | 88,429,815 | 149,840 | 30.09 |
| **Unclassified** | 26,882,850 | 72,075 | 9.15 |
| **Satellite** | 115,341 | 139 | 0.04 |
| **Simple repeats** | 4,621,314 | 95,928 | 1.57 |
| **Low complexity** | 1,400,988 | 25,701 | 0.48 |
| **Total interspersed repeats**^1^ | 150,419,606 | 331,592 | 51.18 |
| **Total** | 156,557,249 | 453,360 | 53.27 |

^1^Repetitive elements for masking genome for gene prediction, except Satellite, Low complexity and Simple repeats. Because this type of repetitive elements are of short length, the probability of appearing in the coding region is high.

**Supplementary Table S9. Functional annotation of predicted genes for *I. indigotica* genome*.***

|  | **Numbers of matching genes** | **Percent of annotated genes (%)** |
| --- | --- | --- |
| **Total** | 30,323 | 100 |
| **Swiss-Prot** | 23,093 | 76.16 |
| **InterPro** | 29,067 | 95.86 |
| **GO** | 26,477 | 87.32 |
| **TrEMBL** | 27,803 | 91.69 |
| **KEGG Pathway** | 8,918 | 29.41 |
| **Annotated**^1^ | 29,522 | 97.36 |
| **Unannotated** | 801 | 2.64 |

^1^At least one match in either of database above.

**Supplementary Table S10. Summary of 22 genomic blocks in *I. indigotica* and *A. thaliana* genomes.**

| **Block** | **AK chromosome** | **At Interval** | **At chromosome** | **Ii Interval** | **Ii chromosome** |
| --- | --- | --- | --- | --- | --- |
| A | 1 | AT1G01010 - AT1G19840 | 1 | Iin08089 - Iin10029 | 1 |
| B | 1 | AT1G19850 - AT1G37130 | 1 | Iin10032 - Iin11551 | 1 |
| C | 1 | AT1G43020 - AT1G56190 | 1 | Iin11600 - Iin12795 | 1 |
| D | 2 | AT1G64670 - AT1G56210 | 1 | Iin02965 - Iin03798 | 5 |
| E | 2 | AT1G64960 - AT1G80950 | 1 | Iin25810 - Iin24122 | 2 |
| F | 3 | AT3G01015 - AT3G25520 | 3 | Iin16502 - Iin14072 | 3 |
| G | 3 | AT2G05170 - AT2G07690 | 2 | Iin13963 - Iin13834 | 3 |
| H | 3 | AT2G10940 - AT2G20900 | 2 | Iin13777 - Iin12816 | 3 |
| I | 4 | AT2G20920 - AT2G31035 | 2 | Iin19861 - Iin18289 | 4 |
| J | 4 | AT2G31040 - AT2G48150 | 2 | Iin18287 - Iin16594 | 4 |
| K-L | 5 | K: AT2G01060 - AT2G05160  L: AT3G25540 - AT3G32960 | 2  3 | Iin01316- Iin01903 | 5 |
| M - N | 5 | AT3G42180 - AT3G63530 | 3 | Iin25887 - Iin27999 | 2 |
| O | 6 | AT4G00026 - AT4G05450 | 4 | Iin08252 - Iin07824 | 6 |
| P | 6 | AT4G12620 - AT4G07390 | 4 | Iin07699 - Iin07133 | 6 |
| Q | 6 | AT5G30510 - AT5G23010 | 5 | Iin01150 - Iin00720 | 5 |
| R | 6 | AT5G23000 - AT5G01010 | 5 | Iin05783 - Iin03904 | 6 |
| S | 7 | AT5G42110 - AT5G32470 | 5 | Ii19892 - Iin20762 | 7 |
| T | 7 | AT4G12700 - AT4G16240 | 4 | Iin20878 - Iin21552 | 7 |
| U | 7 | AT4G16250 - AT4G40100 | 4 | Iin21554 - Iin23810 | 7 |
| V | 8 | AT5G47810 - AT5G42130 | 5 | Iin01912 - Iin02727 | 5 |
| W | 8 | AT5G47820 - AT5G60800 | 5 | Wa: Iin01310 - Iin01151  Wb: Iin07126 - Iin05784 | 5  6 |
| X | 8 | AT5G60805 - AT5G67640 | 5 | Iin00173 - Iin00718 | 5 |

**Supplementary Table S11. Summary of gene family clustering.**

| **Species** | **Total**  **genes** | **Genes in families** | **Family** | **Unclustered genes** | **Unique families** | **Genes per family** |
| --- | --- | --- | --- | --- | --- | --- |
| *A. thaliana* | 27,416 | 23,929 | 20,530 | 3,487 | 147 | 1.17 |
| *B. napus* | 101,040 | 67,243 | 32,131 | 33,797 | 2,791 | 2.09 |
| *B. rapa* | 46,250 | 38,405 | 28,060 | 7,845 | 604 | 1.37 |
| *C. hassleriana* | 26,941 | 23,356 | 15,790 | 3,585 | 353 | 1.48 |
| *C. rubella* | 26,521 | 23,592 | 19,995 | 2,929 | 171 | 1.18 |
| *E. salsugineum* | 26,528 | 23,219 | 19,570 | 3,309 | 149 | 1.18 |
| *I. indigotica* | 30,323 | 24,382 | 18,900 | 5,941 | 896 | 1.29 |
| *R. sativus* | 43,239 | 34,129 | 23,625 | 9,110 | 651 | 1.44 |
| *S. irio* | 49,956 | 37,996 | 21,057 | 11,960 | 1,811 | 1.80 |
| *S. parvula* | 27,132 | 23,537 | 19,345 | 3,595 | 266 | 1.21 |

**Supplemental Table 12. GO enrichment analysis of tandem repeat genes in the *I. indigotica* genome.**

| **GO ID** | **GO Name** | **GO Category** | **FDR** | **P-Value** | **Number of genes** |
| --- | --- | --- | --- | --- | --- |
| GO:0009805 | coumarin biosynthetic process | BP | 6.29E-35 | 4.28E-38 | 120 |
| GO:0080167 | response to karrikin | BP | 3.58E-26 | 5.52E-29 | 222 |
| GO:0009811 | stilbene biosynthetic process | BP | 1.06E-25 | 1.73E-28 | 94 |
| GO:0009625 | response to insect | BP | 2.47E-21 | 5.49E-24 | 87 |
| GO:0050268 | coniferyl-alcohol dehydrogenase activity | MF | 9.65E-20 | 2.54E-22 | 38 |
| GO:0009817 | defense response to fungus, incompatible interaction | BP | 1.10E-19 | 2.95E-22 | 125 |
| GO:0052747 | sinapyl alcohol dehydrogenase activity | MF | 1.65E-19 | 4.71E-22 | 41 |
| GO:0045551 | cinnamyl-alcohol dehydrogenase activity | MF | 1.65E-19 | 4.71E-22 | 41 |
| GO:0045486 | naringenin 3-dioxygenase activity | MF | 5.08E-19 | 1.47E-21 | 51 |
| GO:0042432 | indole biosynthetic process | BP | 5.88E-18 | 2.05E-20 | 52 |
| GO:0080027 | response to herbivore | BP | 2.02E-17 | 7.41E-20 | 53 |
| GO:0031418 | L-ascorbic acid binding | MF | 2.58E-17 | 9.83E-20 | 60 |
| GO:0009815 | 1-aminocyclopropane-1-carboxylate oxidase activity | MF | 1.73E-16 | 6.89E-19 | 35 |
| GO:0005506 | iron ion binding | MF | 1.99E-15 | 8.76E-18 | 133 |
| GO:0102778 | Delta9-tetrahydrocannabinolate synthase activity | MF | 2.46E-15 | 1.09E-17 | 24 |
| GO:0009407 | toxin catabolic process | BP | 5.23E-15 | 2.42E-17 | 41 |
| GO:0020037 | heme binding | MF | 1.26E-14 | 6.05E-17 | 123 |
| GO:0010421 | hydrogen peroxide-mediated programmed cell death | BP | 2.73E-14 | 1.34E-16 | 36 |
| GO:0004364 | glutathione transferase activity | MF | 8.07E-14 | 4.09E-16 | 41 |
| GO:0009809 | lignin biosynthetic process | BP | 9.39E-14 | 4.81E-16 | 128 |
| GO:0009821 | alkaloid biosynthetic process | BP | 1.26E-13 | 6.55E-16 | 63 |
| GO:0102717 | DIMBOA-glucoside oxygenase activity | MF | 1.83E-13 | 9.80E-16 | 19 |
| GO:0120091 | jasmonic acid hydrolase | MF | 2.38E-13 | 1.31E-15 | 39 |
| GO:0102779 | cannabidiolate synthase activity | MF | 3.60E-13 | 2.01E-15 | 22 |
| GO:0080043 | quercetin 3-O-glucosyltransferase activity | MF | 3.90E-12 | 2.45E-14 | 45 |
| GO:0080044 | quercetin 7-O-glucosyltransferase activity | MF | 3.90E-12 | 2.45E-14 | 45 |
| GO:0043295 | glutathione binding | MF | 7.92E-12 | 5.09E-14 | 32 |
| GO:0009624 | response to nematode | BP | 1.35E-11 | 9.09E-14 | 138 |
| GO:0009611 | response to wounding | BP | 1.92E-11 | 1.30E-13 | 359 |
| GO:2000071 | regulation of defense response by callose deposition | BP | 2.13E-11 | 1.46E-13 | 51 |
| GO:0010298 | dihydrocamalexic acid decarboxylase activity | MF | 7.14E-11 | 5.01E-13 | 19 |
| GO:0042937 | tripeptide transmembrane transporter activity | MF | 8.83E-11 | 6.32E-13 | 42 |
| GO:0009682 | induced systemic resistance | BP | 1.16E-10 | 8.47E-13 | 90 |
| GO:0050590 | desacetoxyvindoline 4-hydroxylase activity | MF | 1.16E-10 | 8.46E-13 | 20 |
| GO:0008889 | glycerophosphodiester phosphodiesterase activity | MF | 2.04E-10 | 1.53E-12 | 28 |
| GO:0009718 | anthocyanin-containing compound biosynthetic process | BP | 3.15E-10 | 2.41E-12 | 97 |
| GO:0010112 | regulation of systemic acquired resistance | BP | 3.90E-10 | 3.00E-12 | 45 |
| GO:0071916 | dipeptide transmembrane transporter activity | MF | 4.40E-10 | 3.41E-12 | 36 |
| GO:0042939 | tripeptide transport | BP | 5.16E-10 | 4.04E-12 | 42 |
| GO:0080083 | beta-gentiobiose beta-glucosidase activity | MF | 5.66E-10 | 4.49E-12 | 26 |
| GO:0080054 | low-affinity nitrate transmembrane transporter activity | MF | 8.14E-10 | 6.49E-12 | 32 |
| GO:0033907 | beta-D-fucosidase activity | MF | 1.03E-09 | 8.43E-12 | 26 |
| GO:0031408 | oxylipin biosynthetic process | BP | 1.27E-09 | 1.05E-11 | 39 |
| GO:0046244 | salicylic acid catabolic process | BP | 1.34E-09 | 1.12E-11 | 33 |
| GO:0102483 | scopolin beta-glucosidase activity | MF | 1.40E-09 | 1.17E-11 | 34 |
| GO:0019137 | thioglucosidase activity | MF | 1.50E-09 | 1.26E-11 | 24 |
| GO:0006968 | cellular defense response | BP | 3.01E-09 | 2.59E-11 | 32 |
| GO:0052544 | defense response by callose deposition in cell wall | BP | 3.09E-09 | 2.69E-11 | 61 |
| GO:0033879 | acetylajmaline esterase activity | MF | 3.18E-09 | 2.79E-11 | 18 |
| GO:0045735 | nutrient reservoir activity | MF | 3.85E-09 | 3.41E-11 | 49 |
| GO:0009870 | defense response signaling pathway, resistance gene-dependent | BP | 5.80E-09 | 5.23E-11 | 58 |
| GO:0046256 | 2,4,6-trinitrotoluene catabolic process | BP | 9.76E-09 | 9.69E-11 | 14 |
| GO:0015691 | cadmium ion transport | BP | 1.36E-08 | 1.38E-10 | 30 |
| GO:0102741 | paraxanthine:S-adenosyl-L-methionine 3-N-methyltransferase activity | MF | 1.54E-08 | 1.59E-10 | 21 |
| GO:0102740 | theobromine:S-adenosyl-L-methionine 1-N-methyltransferase activity | MF | 1.54E-08 | 1.59E-10 | 21 |
| GO:0009862 | systemic acquired resistance, salicylic acid mediated signaling pathway | BP | 1.72E-08 | 1.80E-10 | 63 |
| GO:0010193 | response to ozone | BP | 2.30E-08 | 2.43E-10 | 79 |
| GO:0033956 | beta-apiosyl-beta-glucosidase activity | MF | 2.93E-08 | 3.13E-10 | 21 |
| GO:0009816 | defense response to bacterium, incompatible interaction | BP | 3.43E-08 | 3.72E-10 | 121 |
| GO:0042128 | nitrate assimilation | BP | 3.79E-08 | 4.20E-10 | 47 |
| GO:0043201 | response to leucine | BP | 4.25E-08 | 4.73E-10 | 28 |
| GO:0004338 | glucan exo-1,3-beta-glucosidase activity | MF | 4.25E-08 | 4.73E-10 | 28 |
| GO:0071949 | FAD binding | MF | 4.28E-08 | 4.79E-10 | 45 |
| GO:1902289 | negative regulation of defense response to oomycetes | BP | 8.11E-08 | 9.30E-10 | 25 |
| GO:0004867 | serine-type endopeptidase inhibitor activity | MF | 8.11E-08 | 9.30E-10 | 25 |
| GO:0010197 | polar nucleus fusion | BP | 8.22E-08 | 9.47E-10 | 49 |
| GO:0080053 | response to phenylalanine | BP | 8.88E-08 | 1.03E-09 | 26 |
| GO:0008126 | acetylesterase activity | MF | 1.10E-07 | 1.30E-09 | 14 |
| GO:0004121 | cystathionine beta-lyase activity | MF | 1.10E-07 | 1.30E-09 | 14 |
| GO:0047701 | beta-L-arabinosidase activity | MF | 1.26E-07 | 1.51E-09 | 22 |
| GO:0033759 | flavone synthase activity | MF | 1.26E-07 | 1.50E-09 | 25 |
| GO:0006749 | glutathione metabolic process | BP | 1.57E-07 | 1.88E-09 | 48 |
| GO:0002238 | response to molecule of fungal origin | BP | 1.59E-07 | 1.90E-09 | 51 |
| GO:0006071 | glycerol metabolic process | BP | 1.61E-07 | 1.94E-09 | 35 |
| GO:0030275 | LRR domain binding | MF | 1.93E-07 | 2.34E-09 | 47 |
| GO:0042938 | dipeptide transport | BP | 2.52E-07 | 3.11E-09 | 36 |
| GO:0050285 | sinapine esterase activity | MF | 3.07E-07 | 3.82E-09 | 14 |
| GO:0016139 | glycoside catabolic process | BP | 3.85E-07 | 4.87E-09 | 16 |
| GO:0045431 | flavonol synthase activity | MF | 4.60E-07 | 5.83E-09 | 25 |
| GO:0000304 | response to singlet oxygen | BP | 4.63E-07 | 5.90E-09 | 26 |
| GO:0033855 | nicotianamine aminotransferase activity | MF | 5.12E-07 | 6.63E-09 | 11 |
| GO:0080108 | S-alkylthiohydroximate lyase activity | MF | 5.12E-07 | 6.63E-09 | 11 |
| GO:0019863 | IgE binding | MF | 5.12E-07 | 6.61E-09 | 33 |
| GO:0102811 | geraniol 10-hydroxylase activity | MF | 5.96E-07 | 7.77E-09 | 18 |
| GO:0010336 | gibberellic acid homeostasis | BP | 6.53E-07 | 8.56E-09 | 27 |

**Supplemental Table 13. GO enrichment analysis of contracted gene families in the *I. indigotica* genome.**

| **GO ID** | **GO Name** | **GO Category** | **FDR** | **P-Value** | **Number of genes** |
| --- | --- | --- | --- | --- | --- |
| GO:0120029 | proton export across plasma membrane | BP | 3.16E-11 | 2.86E-15 | 11 |
| GO:0007035 | vacuolar acidification | BP | 3.28E-08 | 4.01E-11 | 15 |
| GO:0008553 | proton-exporting ATPase activity, phosphorylative mechanism | MF | 9.97E-08 | 1.85E-10 | 11 |
| GO:0005524 | ATP binding | MF | 3.13E-06 | 1.09E-08 | 158 |
| GO:0000184 | nuclear-transcribed mRNA catabolic process, nonsense-mediated decay | BP | 5.48E-06 | 1.96E-08 | 29 |
| GO:0006614 | SRP-dependent cotranslational protein targeting to membrane | BP | 1.50E-05 | 6.44E-08 | 22 |
| GO:0018105 | peptidyl-serine phosphorylation | BP | 1.97E-05 | 8.75E-08 | 27 |
| GO:0010214 | seed coat development | BP | 2.64E-05 | 1.23E-07 | 28 |
| GO:1990069 | stomatal opening | BP | 3.29E-05 | 1.61E-07 | 17 |
| GO:0010119 | regulation of stomatal movement | BP | 3.64E-05 | 1.81E-07 | 37 |
| GO:0046777 | protein autophosphorylation | BP | 7.51E-05 | 4.28E-07 | 53 |
| GO:0009069 | serine family amino acid metabolic process | BP | 9.38E-05 | 5.56E-07 | 82 |
| GO:0005509 | calcium ion binding | MF | 1.66E-04 | 1.06E-06 | 37 |
| GO:0080110 | sporopollenin biosynthetic process | BP | 2.33E-04 | 1.65E-06 | 10 |
| GO:0000287 | magnesium ion binding | MF | 2.33E-04 | 1.66E-06 | 31 |
| GO:0005516 | calmodulin binding | MF | 2.48E-04 | 1.82E-06 | 37 |
| GO:0006364 | rRNA processing | BP | 2.97E-04 | 2.29E-06 | 43 |
| GO:0004713 | protein tyrosine kinase activity | MF | 3.55E-04 | 2.86E-06 | 30 |
| GO:0071456 | cellular response to hypoxia | BP | 6.55E-04 | 6.16E-06 | 29 |
| GO:0097184 | response to azide | BP | 0.001415617 | 1.45E-05 | 5 |
| GO:0010023 | proanthocyanidin biosynthetic process | BP | 0.001866884 | 2.01E-05 | 11 |
| GO:0080001 | mucilage extrusion from seed coat | BP | 0.001972518 | 2.20E-05 | 9 |
| GO:0052694 | jasmonoyl-isoleucine-12-hydroxylase activity | MF | 0.002524056 | 2.88E-05 | 5 |
| GO:0080133 | midchain alkane hydroxylase activity | MF | 0.002524056 | 2.88E-05 | 5 |
| GO:0098542 | defense response to other organism | BP | 0.002586755 | 3.00E-05 | 159 |
| GO:0007030 | Golgi organization | BP | 0.002599054 | 3.02E-05 | 20 |
| GO:0000281 | mitotic cytokinesis | BP | 0.002618914 | 3.06E-05 | 27 |
| GO:0071347 | cellular response to interleukin-1 | BP | 0.002881821 | 3.43E-05 | 16 |
| GO:0005506 | iron ion binding | MF | 0.003132684 | 3.76E-05 | 27 |
| GO:0004683 | calmodulin-dependent protein kinase activity | MF | 0.003274552 | 4.05E-05 | 10 |
| GO:0048208 | COPII vesicle coating | BP | 0.003301557 | 4.11E-05 | 12 |
| GO:0050302 | indole-3-acetaldehyde oxidase activity | MF | 0.003576059 | 4.53E-05 | 3 |
| GO:0010293 | abscisic aldehyde oxidase activity | MF | 0.003576059 | 4.53E-05 | 3 |
| GO:0005507 | copper ion binding | MF | 0.003641358 | 4.65E-05 | 26 |
| GO:0048480 | stigma development | BP | 0.00392916 | 5.21E-05 | 5 |
| GO:0009931 | calcium-dependent protein serine/threonine kinase activity | MF | 0.003940336 | 5.26E-05 | 8 |
| GO:0020037 | heme binding | MF | 0.004394318 | 5.94E-05 | 25 |
| GO:0019083 | viral transcription | BP | 0.004701802 | 6.53E-05 | 21 |
| GO:0010037 | response to carbon dioxide | BP | 0.004772669 | 6.65E-05 | 9 |
| GO:0018685 | alkane 1-monooxygenase activity | MF | 0.004813101 | 6.82E-05 | 5 |
| GO:0030435 | sporulation resulting in formation of a cellular spore | BP | 0.005047933 | 7.33E-05 | 24 |
| GO:0022843 | voltage-gated cation channel activity | MF | 0.005047933 | 7.36E-05 | 9 |
| GO:0071949 | FAD binding | MF | 0.005047933 | 7.37E-05 | 12 |
| GO:0018479 | benzaldehyde dehydrogenase (NAD+) activity | MF | 0.005080758 | 7.54E-05 | 4 |
| GO:0016175 | superoxide-generating NADPH oxidase activity | MF | 0.005690913 | 8.78E-05 | 5 |
| GO:0036180 | filamentous growth of a population of unicellular organisms in response to biotic stimulus | BP | 0.006091737 | 9.51E-05 | 8 |
| GO:0016477 | cell migration | BP | 0.006340759 | 9.99E-05 | 38 |
| GO:0042532 | negative regulation of tyrosine phosphorylation of STAT protein | BP | 0.006943108 | 1.11E-04 | 4 |
| GO:0030506 | ankyrin binding | MF | 0.006943108 | 1.11E-04 | 4 |
| GO:0070330 | aromatase activity | MF | 0.006943108 | 1.12E-04 | 5 |
| GO:0031369 | translation initiation factor binding | MF | 0.007439951 | 1.22E-04 | 11 |
| GO:0030587 | sorocarp development | BP | 0.008519602 | 1.45E-04 | 26 |
| GO:0042803 | protein homodimerization activity | MF | 0.008519602 | 1.44E-04 | 63 |
| GO:1900436 | positive regulation of filamentous growth of a population of unicellular organisms in response to starvation | BP | 0.008976176 | 1.58E-04 | 4 |
| GO:0043279 | response to alkaloid | BP | 0.009101922 | 1.61E-04 | 14 |
| GO:0010025 | wax biosynthetic process | BP | 0.009739081 | 1.74E-04 | 11 |
| GO:0055114 | oxidation-reduction process | BP | 0.009790961 | 1.75E-04 | 77 |
| GO:0046686 | response to cadmium ion | BP | 0.010048341 | 1.81E-04 | 53 |
| GO:0006119 | oxidative phosphorylation | BP | 0.010680325 | 1.94E-04 | 15 |
| GO:0007291 | sperm individualization | BP | 0.010689298 | 1.95E-04 | 6 |
| GO:0005217 | intracellular ligand-gated ion channel activity | MF | 0.010689298 | 1.95E-04 | 6 |
| GO:0015079 | potassium ion transmembrane transporter activity | MF | 0.011532275 | 2.13E-04 | 15 |
| GO:0140027 | establishment of contractile vacuole localization | BP | 0.011549224 | 2.17E-04 | 4 |
| GO:0046298 | 2,4-dichlorobenzoate catabolic process | BP | 0.011549224 | 2.19E-04 | 3 |
| GO:0060069 | Wnt signaling pathway, regulating spindle positioning | BP | 0.011549224 | 2.19E-04 | 3 |
| GO:0009617 | response to bacterium | BP | 0.011549224 | 2.16E-04 | 114 |
| GO:0010425 | DNA methylation on cytosine within a CNG sequence | BP | 0.011549224 | 2.19E-04 | 3 |
| GO:2000672 | negative regulation of motor neuron apoptotic process | BP | 0.011549224 | 2.19E-04 | 3 |
| GO:0016463 | zinc transmembrane transporter activity, phosphorylative mechanism | MF | 0.011549224 | 2.19E-04 | 3 |
| GO:0008551 | cadmium transmembrane transporter activity, phosphorylative mechanism | MF | 0.011549224 | 2.19E-04 | 3 |
| GO:0050268 | coniferyl-alcohol dehydrogenase activity | MF | 0.012113855 | 2.32E-04 | 7 |
| GO:0099094 | ligand-gated cation channel activity | MF | 0.012113855 | 2.32E-04 | 7 |
| GO:0009651 | response to salt stress | BP | 0.012255395 | 2.35E-04 | 94 |
| GO:0017000 | antibiotic biosynthetic process | BP | 0.01333583 | 2.62E-04 | 17 |
| GO:0010152 | pollen maturation | BP | 0.014570026 | 2.94E-04 | 11 |
| GO:0003779 | actin binding | MF | 0.01461459 | 2.96E-04 | 21 |
| GO:0010197 | polar nucleus fusion | BP | 0.014697095 | 2.98E-04 | 12 |
| GO:1990090 | cellular response to nerve growth factor stimulus | BP | 0.014927092 | 3.05E-04 | 9 |
| GO:0098657 | import into cell | BP | 0.015210354 | 3.12E-04 | 47 |

**Supplemental Table 14. Genes involved in terpenoids and sterols biosynthetic pathways identified in *I. indigotica* genome.**

| **KO number** | **EC number** | **Description** | **Abbreviation** | **Copy number** | **Gene ID** |
| --- | --- | --- | --- | --- | --- |
| **K01662** | **2.2.1.7** | 1-deoxy-D-xylulose-5-phosphate synthase | DXS | 3 | Iin04779 Iin14581 Iin21466 |
| **K00099** | **1.1.1.267** | 1-deoxy-D-xylulose-5-phosphate reductoisomerase | DXR | 1 | Iin00506 |
| **K00991** | **2.7.7.60** | 2-C-methyl-D-erythritol 4-phosphate cytidylyltransferase | MCT | 1 | Iin01765 |
| **K00919** | **2.7.1.148** | 4-diphosphocytidyl-2-C-methyl-D-erythritol kinase | CMK | 1 | Iin18833 |
| **K01770** | **4.6.1.12** | 2-C-methyl-D-erythritol 2,4-cyclodiphosphate synthase | MCS | 1 | Iin03714 |
| **K03526** | **1.17.7.1**  **1.17.7.3** | 4-hydroxy-3-methylbut-2-en-1-yl diphosphate synthase | HDS | 1 | Iin05804 |
| **K03527** | **1.17.7.4** | 4-hydroxy-3-methylbut-2-en-1-yl diphosphate reductase | HDR | 1 | Iin23455 |
| **K01823** | **5.3.3.2** | isopentenyl-diphosphate Delta-isomerase | IDI | 1 | Iin16425 |
| **K13789** | **[2.5.1.1](https://www.kegg.jp/dbget-bin/www_bget?ec:2.5.1.1)**  **[2.5.1.10](https://www.kegg.jp/dbget-bin/www_bget?ec:2.5.1.10)**  **[2.5.1.29](https://www.kegg.jp/dbget-bin/www_bget?ec:2.5.1.29)** | geranylgeranyl diphosphate synthase | GGPS | 9 | Iin13052  Iin13054  Iin14736  Iin14738  Iin14739  Iin15292  Iin19517  Iin23689  Iin23973 |
| **K00626** | **2.3.1.9** | acetyl-CoA C-acetyltransferase | ACAT | 2 | Iin01277 Iin01919 |
| **K01641** | **2.3.3.10** | hydroxymethylglutaryl-CoA synthase | HMCS | 1 | Iin07607 |
| **K00021** | **1.1.1.34**  **1.1.1.88** | hydroxymethylglutaryl-CoA reductase | HMCR | 3 | Iin13206  Iin13208 Iin24457 |
| **K00869** | **2.7.1.36** | mevalonate kinase | MK | 1 | Iin01079 |
| **K00938** | **2.7.4.2** | phosphomevalonate kinase | PMK | 1 | Iin11095 |
| **K01597** | **4.1.1.33** | diphosphomevalonate decarboxylase | MVD | 1 | Iin27116 |
| **K00787** | **[2.5.1.1](https://www.kegg.jp/dbget-bin/www_bget?ec:2.5.1.1)**  **[2.5.1.10](https://www.kegg.jp/dbget-bin/www_bget?ec:2.5.1.10)** | farnesyl diphosphate synthase | FDPS | 1 | Iin01915 |
| **K00801** | **2.5.1.21** | farnesyl-diphosphate farnesyltransferase | FDFT | 2 | Iin23487  Iin23488 |
| **K00511** | **1.14.14.17** | squalene monooxygenase | SQLE | 9 | Iin00819  Iin00820  Iin00821  Iin02002  Iin02003  Iin02004  Iin03169  Iin19618  Iin23772 |
| **K01853** | **5.4.99.8** | cycloartenol synthase | CAS | 2 | Iin13831  Iin15607 |
| **K00559** | **2.1.1.41** | sterol 24-C-methyltransferase | SMT1 | 1 | Iin04963 |
| **K14423** | **1.14.18.10** | plant 4,4-dimethylsterol C-4alpha-methyl-monooxygenase | SMO1 | 2 | Iin07642  Iin22242 |
| **K23558** | **1.1.1.418** | plant 3beta-hydroxysteroid-4alpha-carboxylate 3-dehydrogenase | HSD | 2 | Iin11888  Iin19016 |
| **K08246** | **[5.5.1.9](https://www.kegg.jp/dbget-bin/www_bget?ec:5.5.1.9)** | cycloeucalenol cycloisomerase | CCI | 2 | Iin06986  Iin19336 |
| **K05917** | **[1.14.14.154](https://www.kegg.jp/dbget-bin/www_bget?ec:1.14.14.154)**  **[1.14.15.36](https://www.kegg.jp/dbget-bin/www_bget?ec:1.14.15.36)** | sterol 14alpha-demethylase | SAD | 1 | Iin09288 |
| **K00222** | **1.3.1.70** | Delta14-sterol reductase | DSR1 | 1 | Iin26980 |
| **K01824** | **5.3.3.5** | cholestenol Delta-isomerase | CDI | 1 | Iin10046 |
| **K08242** | **2.1.1.143** | 24-methylenesterol C-methyltransferase | SMT2 | 1 | Iin10073 |
| **K14424** | **1.14.18.11** | plant 4alpha-monomethylsterol monooxygenase | SMO2 | 2 | Iin08862  Iin18475 |
| **K00227** | **[1.14.19.20](https://www.kegg.jp/dbget-bin/www_bget?ec:1.14.19.20)** | Delta7-sterol 5-desaturase | DSD | 1 | Iin16444 |
| **K00213** | **[1.3.1.21](https://www.kegg.jp/dbget-bin/www_bget?ec:1.3.1.21)** | 7-dehydrocholesterol reductase | DCSR7 | 1 | Iin12191 |
| **K09828** | **[1.3.1.72](https://www.kegg.jp/dbget-bin/www_bget?ec:1.3.1.72)**  **1.3.1.-** | Delta24-sterol reductase | DSR2 | 2 | Iin12177  Iin14779 |

**Supplemental Table 15. Genes involved in lignans and flavonoids biosynthetic pathways identified in *I. indigotica* genome.**

| **KO number** | **EC number** | **Description** | **Abbreviation** | **Copy number** | **Gene ID** |
| --- | --- | --- | --- | --- | --- |
| **K10775** | **4.3.1.24** | phenylalanine ammonia-lyase | PAL | 3 | Iin15715  Iin17685  Iin27014 |
| **K00487** | **1.14.14.91** | cinnamate 4-hydroxylase | C4H | 6 | Iin18350  Iin18353  Iin18354  Iin29709  Iin29712  Iin29713 |
| **K01904** | **6.2.1.2** | 4-coumarate-CoA ligase | 4CL | 6 | Iin12363  Iin14612  Iin14613  Iin25786  Iin28525  Iin29393 |
| **K00660** | **2.3.1.74** | chalcone synthase | CHS | 3 | Iin01239  Iin01240  Iin04984 |
| **K01859** | **5.5.1.6** | chalcone isomerase | CHI | 2 | Iin00040  Iin27202 |
| **K13077** | **1.14.20.5** | flavone synthase I | FNSI | 2 | Iin19302  Iin26795 |
| **K13065** | **2.3.1.133** | shikimate O-hydroxycinnamoyltransferase | HCT | 2 | Iin06079  Iin13004 |
| **K09754** | **1.14.14.96** | 5-O-(4-coumaroyl)-D-quinate 3'-monooxygenase | C3’H | 1 | Iin17255 |
| **K00588** | **2.1.1.104** | caffeoyl-CoA O-methyltransferase | CCoAOMT | 5 | Iin10582  Iin22645  Iin23419  Iin25370  Iin25371 |
| **K09753** | **1.2.1.44** | cinnamoyl-CoA reductase | CCR | 1 | Iin24104 |
| **K00083** | **1.1.1.195** | cinnamyl-alcohol dehydrogenase | CAD | 9 | Iin14823  Iin19761  Iin23442  Iin23794  Iin23795  Iin23796  Iin23889  Iin24841  Iin29160 |
| **-** | **-** | Dirigent protein | DIR | 21 | Iin02771  Iin06887  Iin07549  Iin10303  Iin10304  Iin12672  Iin14298  Iin15393  Iin15394  Iin17412  Iin18531  Iin19845  Iin19874  Iin20509  Iin21107  Iin22345  Iin22360  Iin25914  Iin27211  Iin27522  Iin28172 |
| **K21568** | **[1.23.1.1](https://www.kegg.jp/dbget-bin/www_bget?ec:1.23.1.1)**  **[1.23.1.2](https://www.kegg.jp/dbget-bin/www_bget?ec:1.23.1.2)**  **[1.23.1.3](https://www.kegg.jp/dbget-bin/www_bget?ec:1.23.1.3)**  **[1.23.1.4](https://www.kegg.jp/dbget-bin/www_bget?ec:1.23.1.4)** | pinoresinol/lariciresinol reductase | PLR | 3 | Iin11112  Iin11113  Iin21123 |
| **K19650** | **1.1.1.331** | secoisolariciresinol dehydrogenase | SDH | 2 | Iin12437  Iin26834 |

**Supplemental Table 16. Genes involved in indole alkaloids biosynthetic pathways identified in *I. indigotica* genome.**

| **KO number** | **EC number** | **Description** | **Abbreviation** | **Copy number** | **Gene ID** |
| --- | --- | --- | --- | --- | --- |
| **K01593** | **[4.1.1.28](https://www.kegg.jp/dbget-bin/www_bget?ec:4.1.1.28)**  **[4.1.1.105](https://www.kegg.jp/dbget-bin/www_bget?ec:4.1.1.105)** | aromatic-L-amino-acid/L-tryptophan decarboxylase | DDC | 2 | Iin12872  Iin22896 |
| **K04103** | **[4.1.1.74](https://www.kegg.jp/dbget-bin/www_bget?ec:4.1.1.74)** | indole pyruvate decarboxylase | IPDC | 3 | Iin03877  Iin06394  Iin15840 |
| **K16903** | **2.6.1.99** | L-tryptophan---pyruvate aminotransferase | TAA | 3 | Iin10350  Iin22479  Iin25059 |
| **K11816** | **1.14.13.168** | indole-3-pyruvate monooxygenase | YUCCA | 11 | Iin00954  Iin02515  Iin04775  Iin08555  Iin08597  Iin10164  Iin12065  Iin18074  Iin21070  Iin22904  Iin23276 |
| **K11182**  **K11183** | **1.14.14.156** | tryptophan N-monooxygenase | CYP79B | 2 | Iin19688  Iin23829 |
| **K11868** | **4.99.1.6** | indoleacetaldoxime dehydratase | CYP71A13 | 1 | Iin18328 |
| **K01501** | **3.5.5.1** | nitrilase | NIT | 4 | Iin01346  Iin26007  Iin26009  Iin26015 |
| **K18848** | **[2.1.1.278](https://www.kegg.jp/dbget-bin/www_bget?ec:2.1.1.278)** | indole-3-acetate O-methyltransferase | IAMT | 1 | Iin06360 |
| **-** | **2.4.1.220** | indoxyl-UDPG glucosyltransferase | IUGT | 1 | Iin08137 |
| **K01667** | **4.1.99.1** | tryptophanase | tnaA | 2 | Iin03581  Iin09341 |
| **-** | **-** | flavin-dependent monooxygenase | FMO | 2 | Iin09976  Iin09977 |

**Figures**


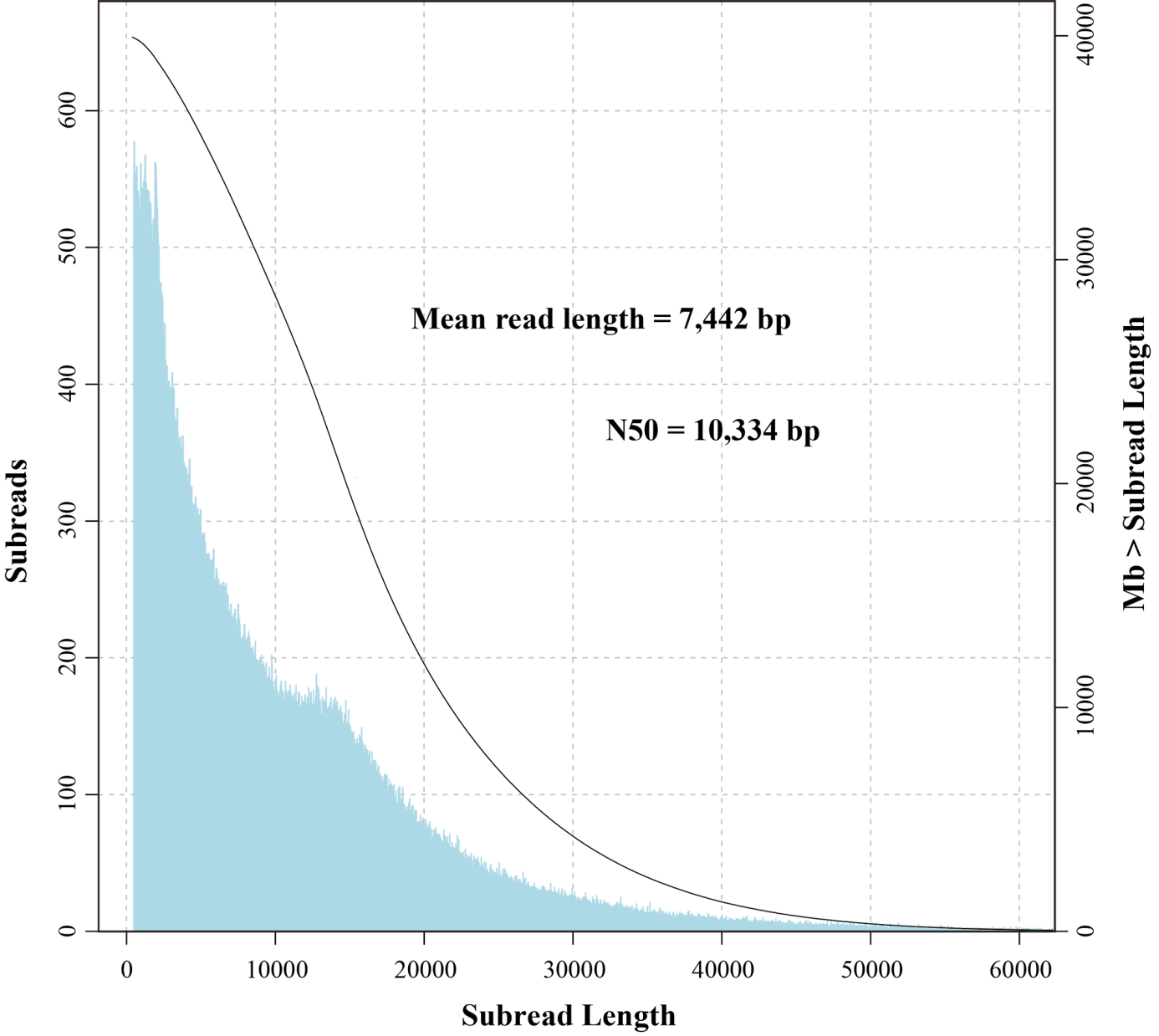


**Supplementary Figure S1. Histogram of length distribution of raw *I. indigotica* PacBio subreads.**


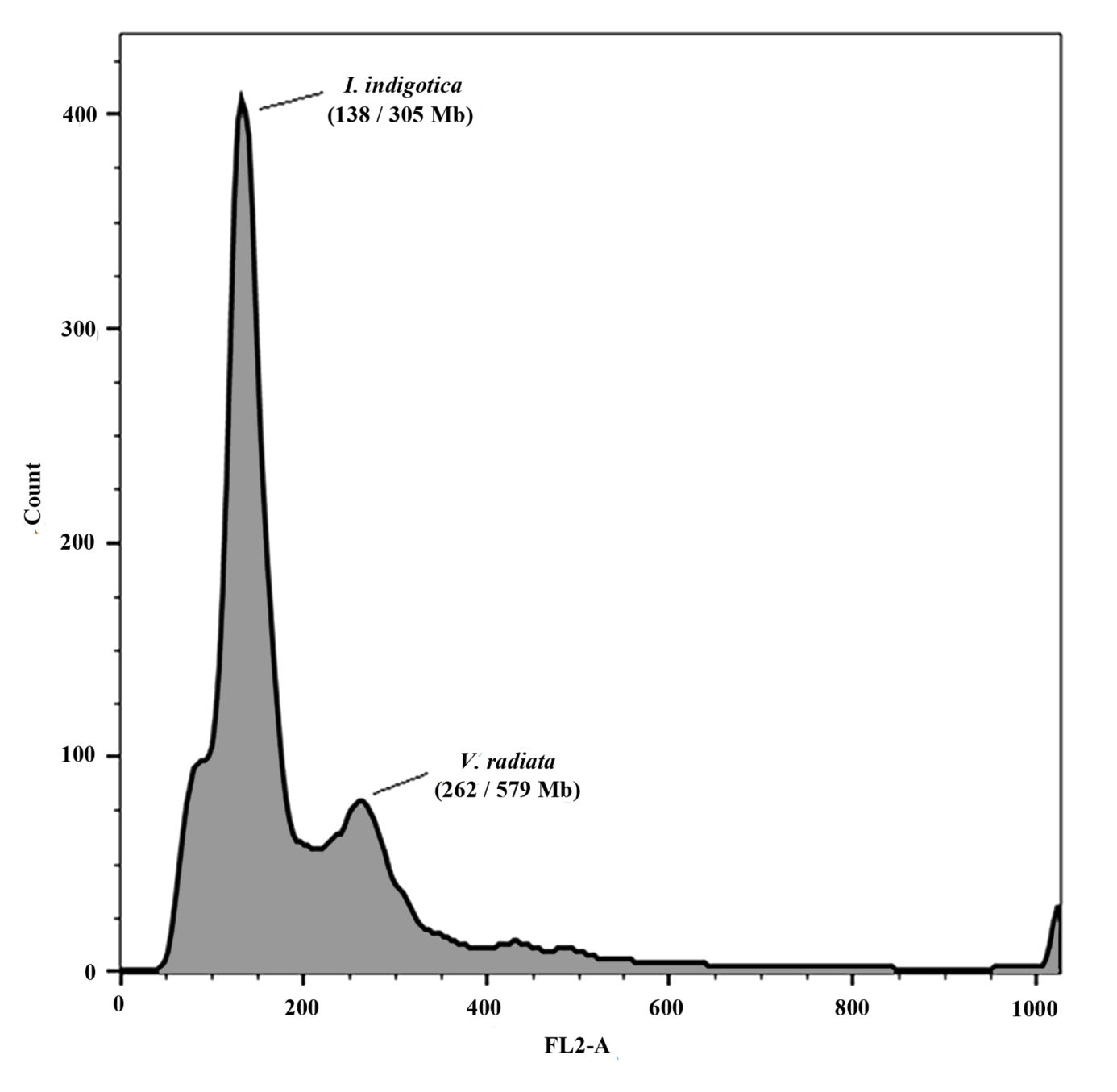


**Supplementary Figure S2. Flow cytometric analysis results.** The genome size of *Vigna radiata* is estimated 579 Mb previously, and the peak value is 262, while *I. indigotica,* which peak value is 138, and estimated its genome size to be 305 Mb.


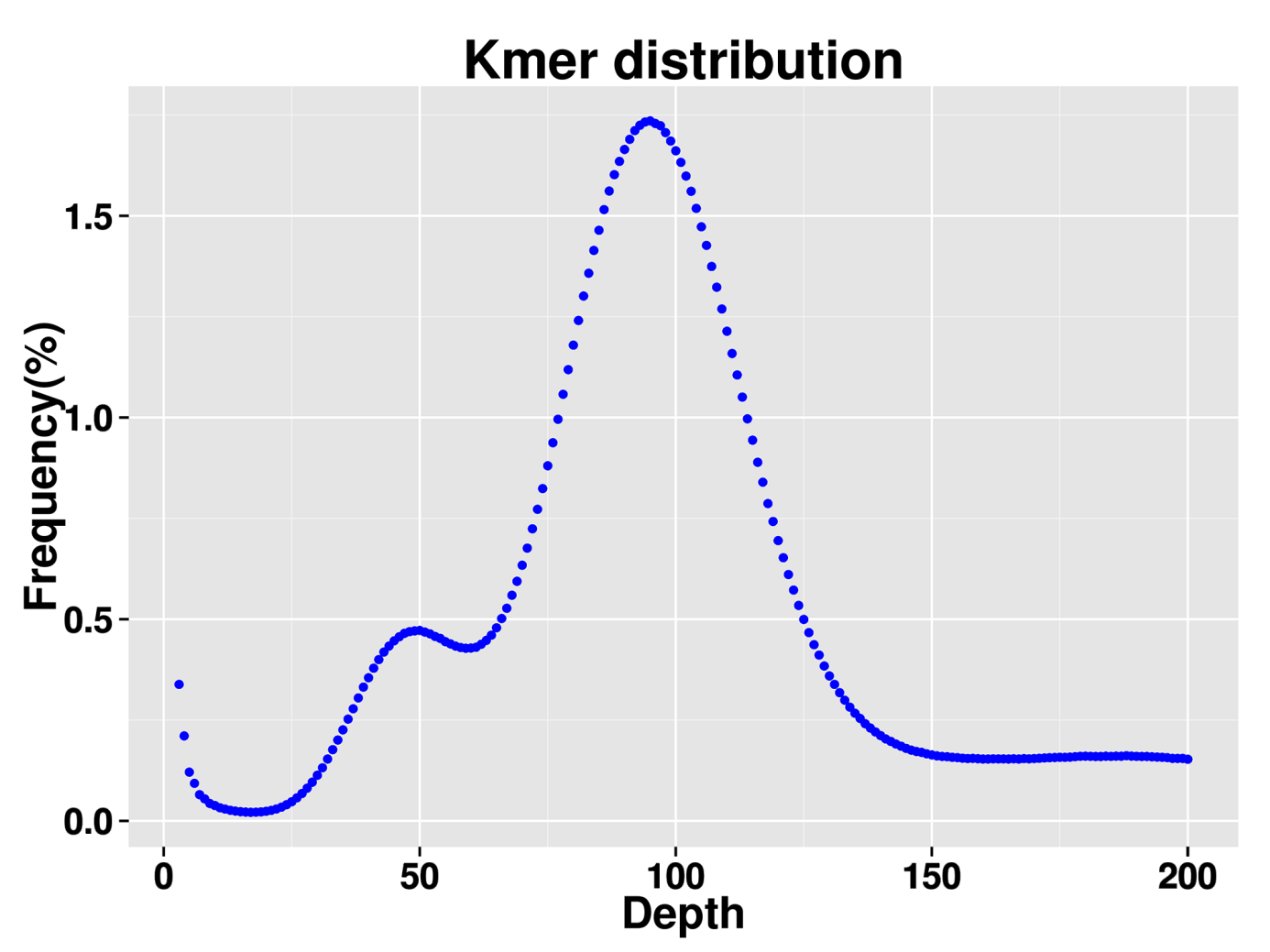


**Supplementary Figure S3.** **Estimation of *I. indigotica* genome size by K-mer analysis.** The figure shows the frequency of 19 k-mers, which are 19 bp sequences from clean reads of short-insert-size libraries. We identified 26,559,599,210 K-mers and the peak of K-mer depth is 94. Genome size can be estimated as (total K-mer number)/(the volume peak). The genome size of *I. indigotica* was thus estimated as 279.90 Mb.


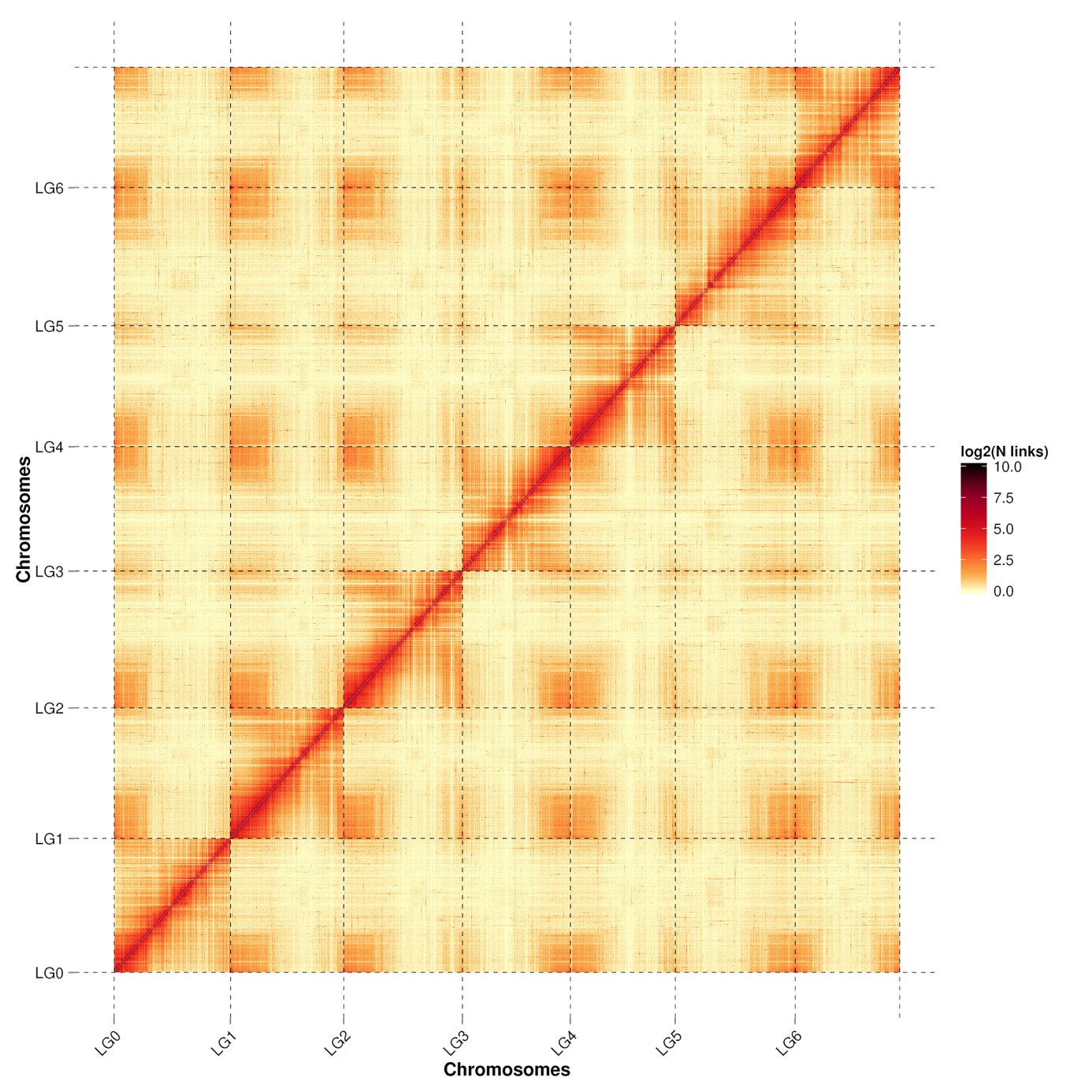


**Supplementary Figure** S**4.** **Hi-C map of the *I. indigotica* genome showing genome-wide all-by-all interactions.** The map shows a high resolution of individual chromosomes that are scaffolded and assembled independently.


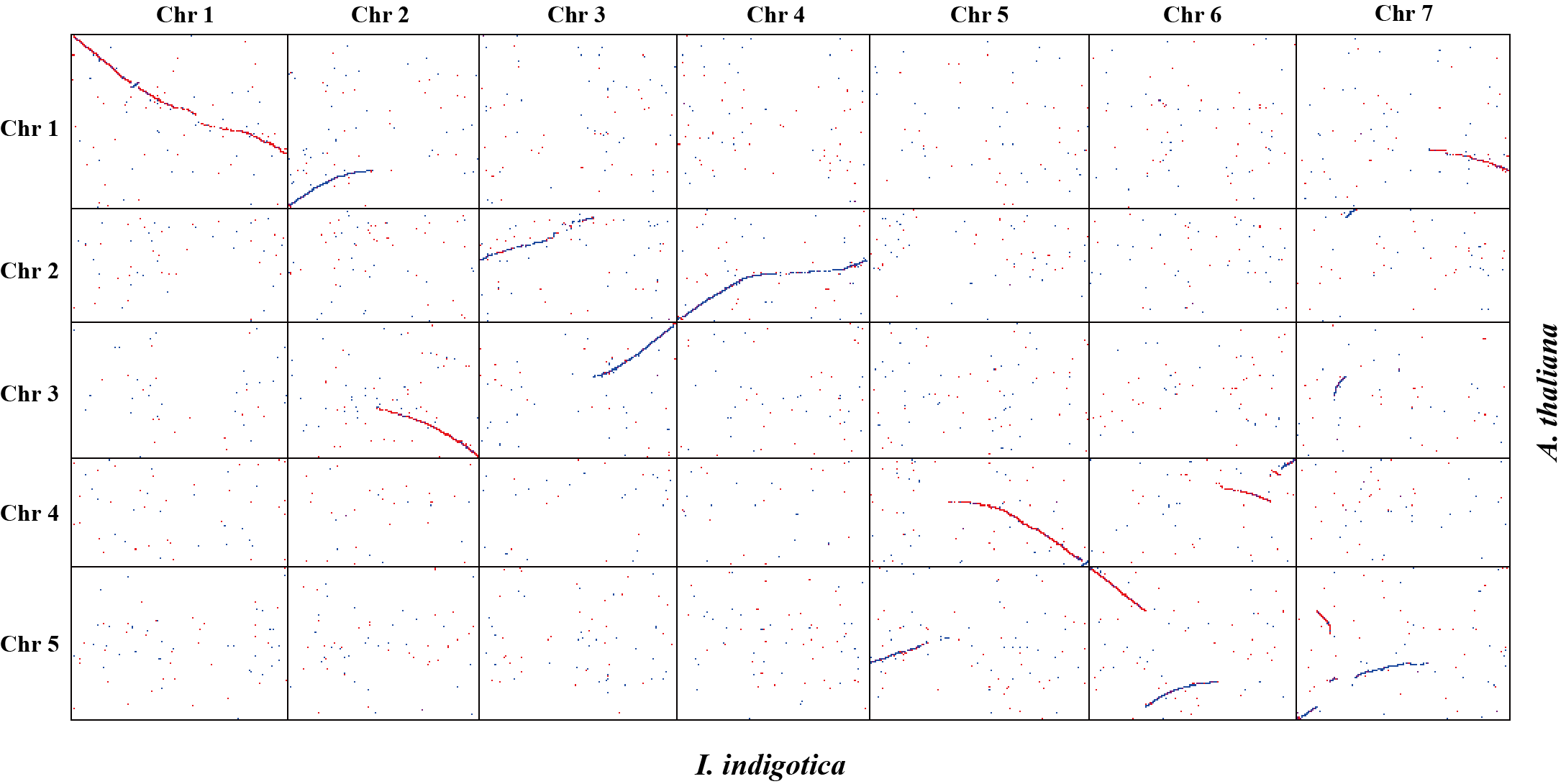


**Supplementary Figure S5. Dot plot illustrating the comparative analysis between *I. indigotica* and *A. thaliana* genomes.** The dots represent the genome sequences’ synteny detected by last v946.


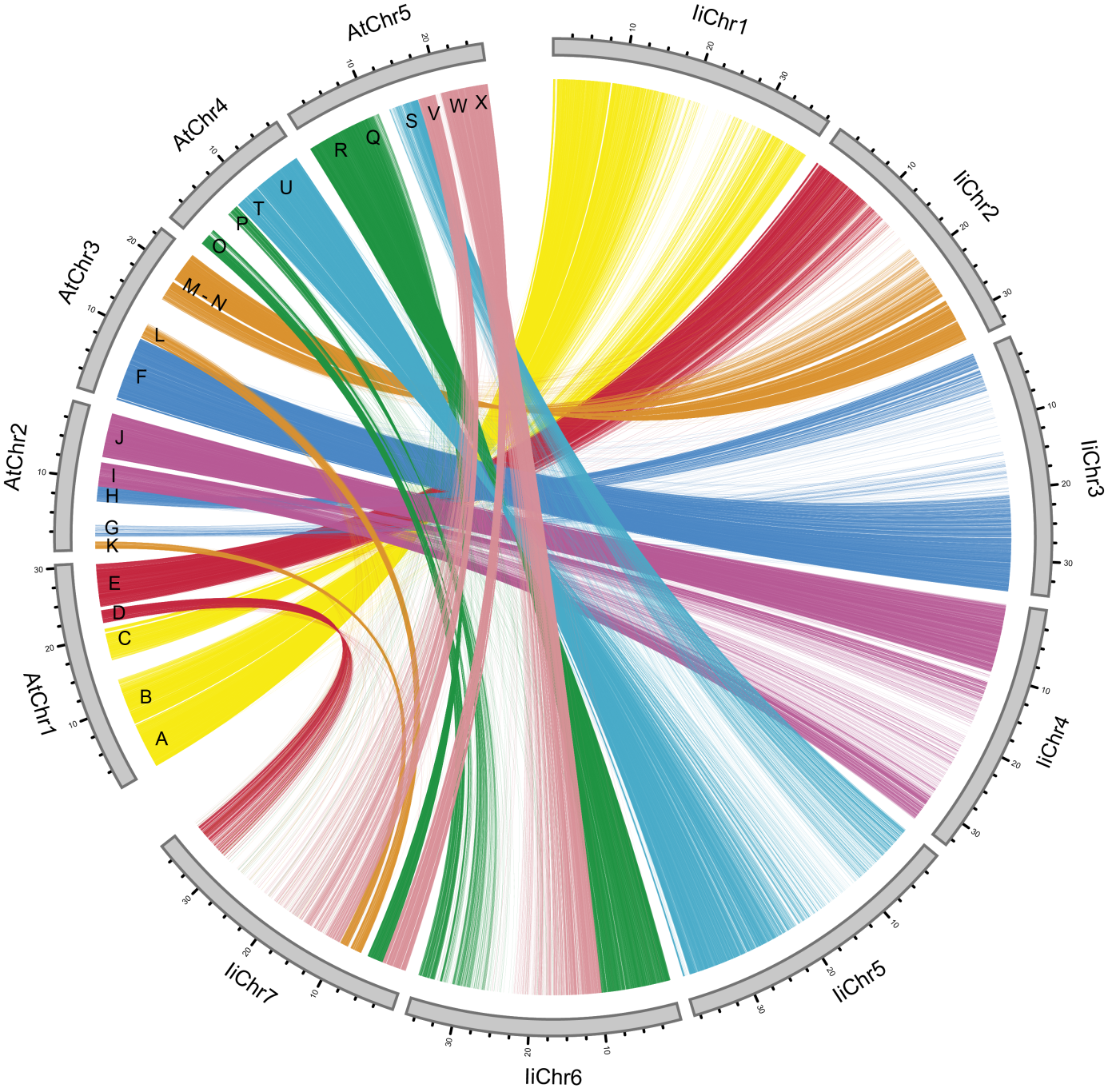


**Supplementary Figure S6. Circos plot of the *I. indigotica* genome with *A. thaliana* 5 chromosomes.** Circos plot presenting the relationships of the GBs in *A. thaliana* and *I. indigotica* chromosomes, the links connecting them are depicted with colors.


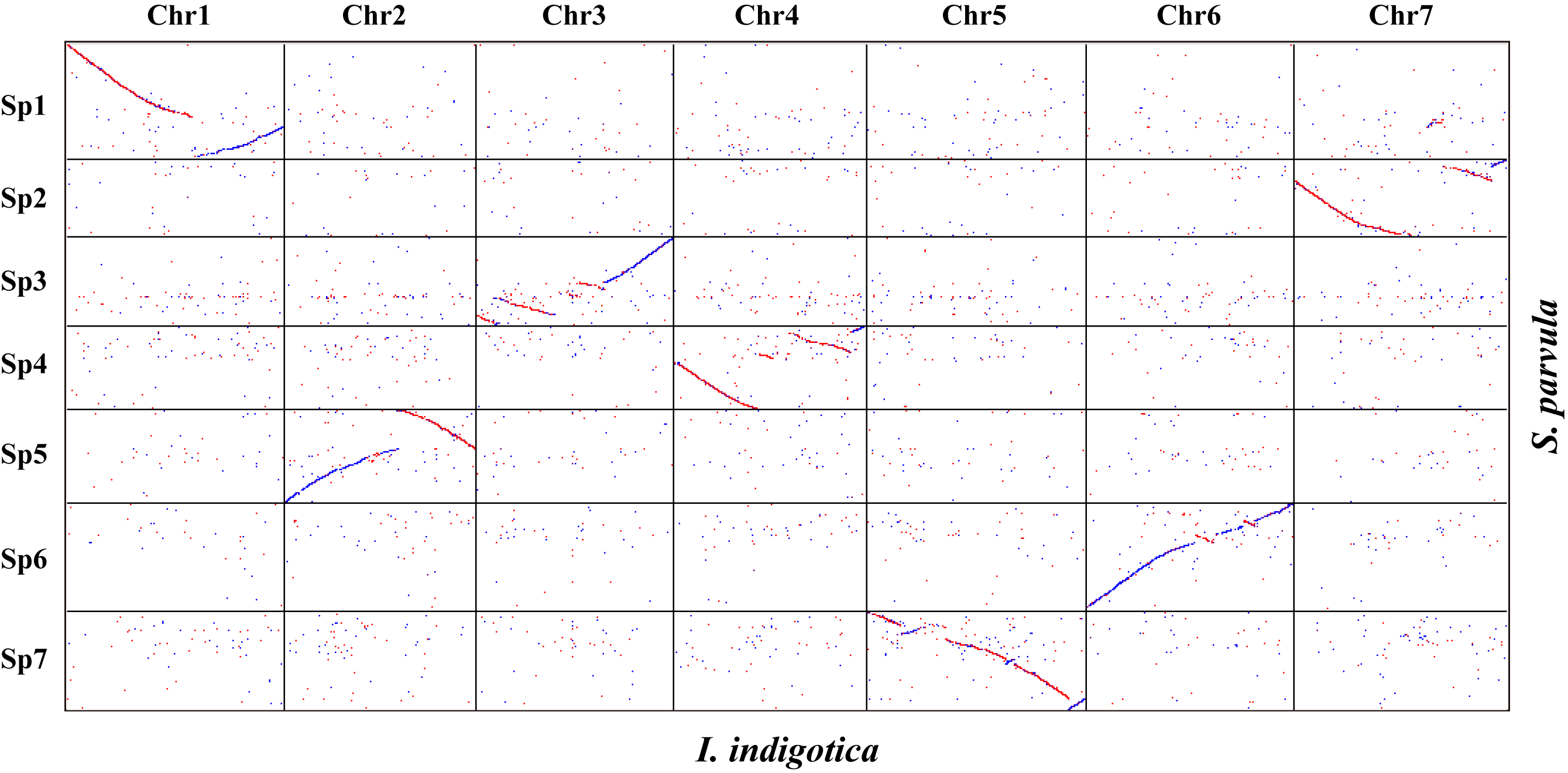


**Supplementary Figure S7. Dot plot illustrating the comparative analysis between *I. indigotica* and *S. parvula* genomes.** The dots represent the genome sequences’ synteny detected by last v946.


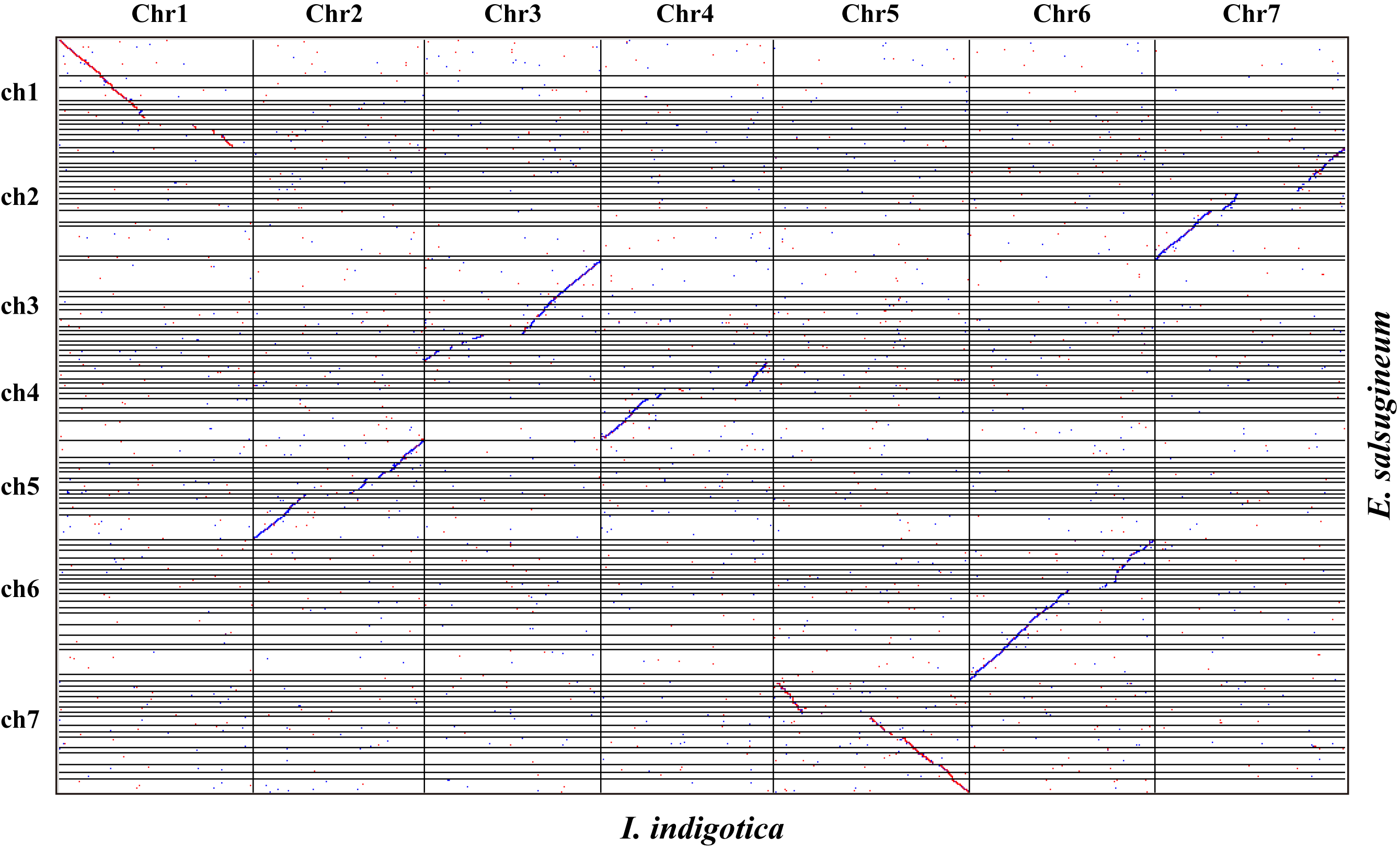


**Supplementary Figure S8. Dot plot illustrating the comparative analysis between *I. indigotica* and *E. salsugineum* genomes.** The dots represent the genome sequences’ synteny detected by last v946.


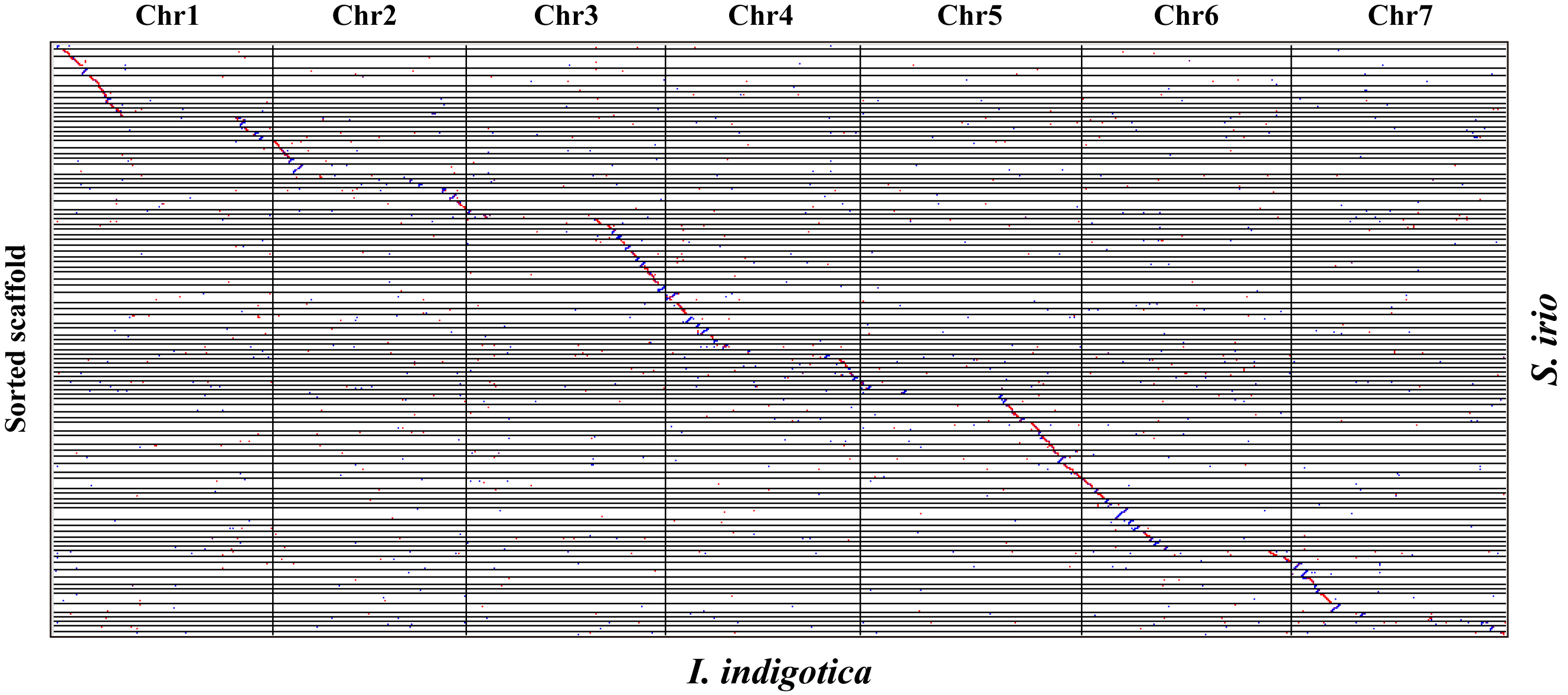


**Supplementary Figure S9. Dot plot illustrating the comparative analysis between *I. indigotica* and *S. irio* genomes.** The dots represent the genome sequences’ synteny detected by last v946.


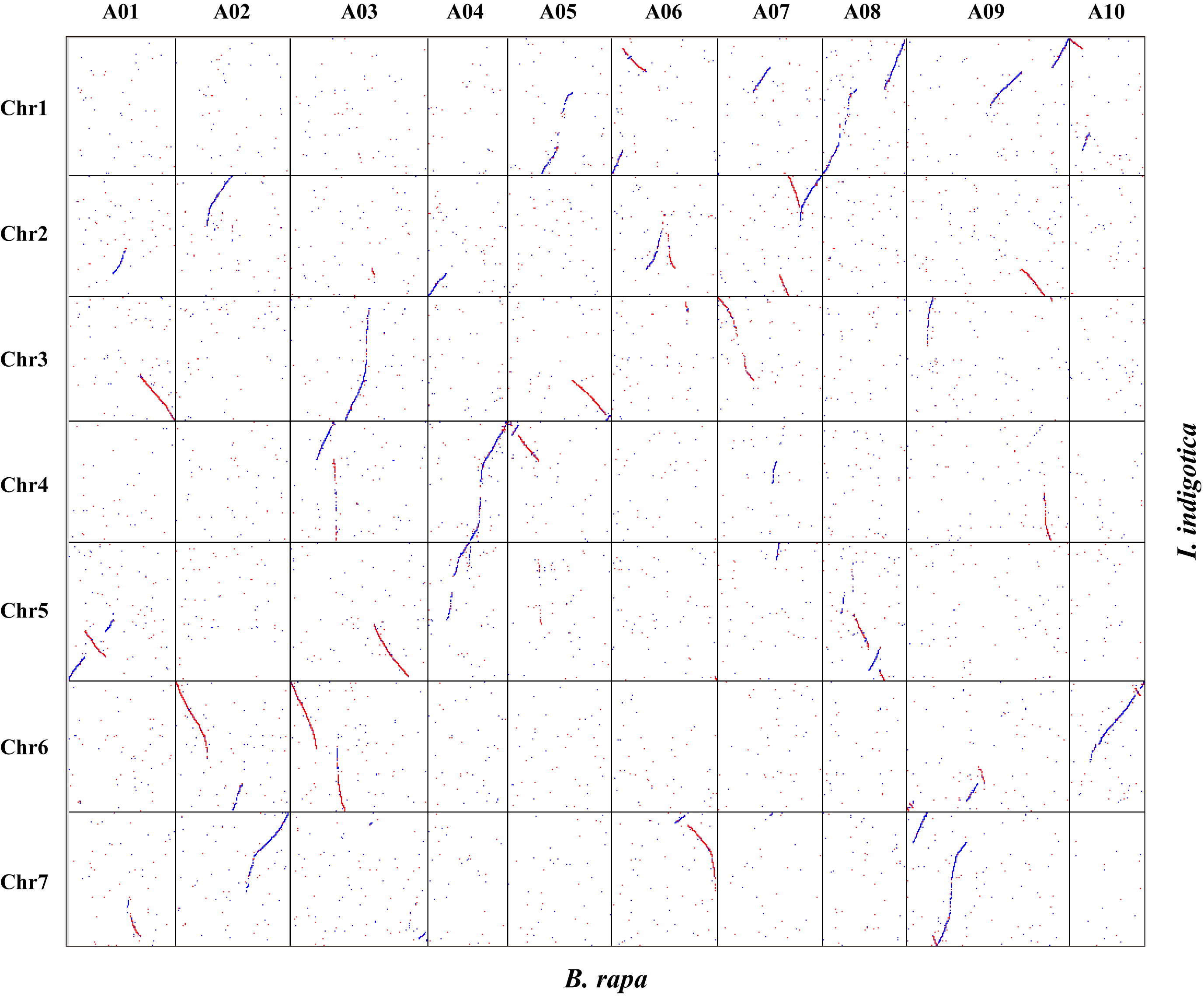


**Supplementary Figure S10. Dot plot illustrating the comparative analysis between *I. indigotica* and *B. rapa* genomes.** The dots represent the genome sequences’ synteny detected by last v946.
